# Genome-resolved evidence for functionally redundant communities and novel nitrogen fixers in the deyin-1 hydrothermal field, Mid-Atlantic Ridge

**DOI:** 10.1186/s40168-021-01202-x

**Published:** 2022-01-19

**Authors:** Jie Pan, Wei Xu, Zhichao Zhou, Zongze Shao, Chunming Dong, Lirui Liu, Zhuhua Luo, Meng Li

**Affiliations:** 1 Archaeal Biology Center, Institute for Advanced Study, Shenzhen University, Shenzhen, Guangdong People’s Republic of China; 2 Key Laboratory of Marine Biogenetic Resources, Third Institute of Oceanography, Ministry of Natural Resources, Fujian Xiamen, People’s Republic of China; 3 Department of Bacteriology, University of Wisconsin-Madison, Madison, WI 53706 USA; 4 School of Marine Sciences, Nanjing University of Information Science & Technology, 210044 Nanjing, People’s Republic of China

**Keywords:** Microbial community, Metabolisms, Nitrogen fixation, Functional redundancy, Deep-sea hydrothermal field, Metabolic plasticity, Deyin-1

## Abstract

**Background:**

Deep-sea hydrothermal vents represent unique ecosystems that redefine our understanding of the limits of life. They are widely distributed in deep oceans and typically form along mid-ocean ridges. To date, the hydrothermal systems in the Mid-Atlantic Ridge south of 14°S remain barely explored, limiting our understanding of the microbial community in this distinct ecosystem. The Deyin-1 is a newly discovered hydrothermal field in this area. By applying the metagenomic analysis, we aim at gaining much knowledge of the biodiversity and functional capability of microbial community inhabiting this field.

**Results:**

In the current study, 219 metagenomic assembled genomes (MAGs) were reconstructed, unveiling a diverse and variable community dominated by Bacteroidetes, Nitrospirae, Alpha-, Delta-, and Gammaproteobacteria in the active and inactive chimney samples as well as hydrothermal oxide samples. Most of these major taxa were potentially capable of using reduced sulfur and hydrogen as primary energy sources. Many members within the major taxa exhibited potentials of metabolic plasticity by possessing multiple energy metabolic pathways. Among these samples, different bacteria were found to be the major players of the same metabolic pathways, further supporting the variable and functionally redundant community in situ. In addition, a high proportion of MAGs harbored the genes of carbon fixation and extracellular carbohydrate-active enzymes, suggesting that both heterotrophic and autotrophic strategies could be essential for their survival. Notably, for the first time, the genus *Candidatus* Magnetobacterium was shown to potentially fix nitrogen, indicating its important role in the nitrogen cycle of inactive chimneys. Moreover, the metabolic plasticity of microbes, diverse and variable community composition, and functional redundancy of microbial communities may represent the adaptation strategies to the geochemically complex and fluctuating environmental conditions in deep-sea hydrothermal fields.

**Conclusions:**

This represents the first assembled-genome-based investigation into the microbial community and metabolism of a hydrothermal field in the Mid-Atlantic Ridge south of 14°S. The findings revealed that a high proportion of microbes could benefit from simultaneous use of heterotrophic and autotrophic strategies in situ. It also presented novel members of potential diazotrophs and highlighted the metabolic plasticity and functional redundancy across deep-sea hydrothermal systems.

Video abstract

**Supplementary Information:**

The online version contains supplementary material available at 10.1186/s40168-021-01202-x.

## Background

Deep-sea hydrothermal vents are one of the most extreme environments on Earth, with temperatures ranging from tens of degrees to more than 670 Kelvin (400°C) and water depths of ~200 to ~5000 m [1]. Since the first discovery of “thermal springs” on the Galapagos Ridge in 1977 [2], the exploration of deep-sea hydrothermal vents has never ceased. Numerous studies have investigated microbial communities inhabiting deep-sea hydrothermal systems, elucidating the unique diversity and metabolism of microorganisms in the biogeochemical cycles of deep sea [3–9].

Deep-sea hydrothermal vents are energy hot spots in the ocean, supporting abundant chemolithoautotrophic microorganisms that fix inorganic carbon to organic carbon with the energy from redox chemical reactions [10–13]. By providing substantial primary production, these microorganisms transfer the chemical elements and energy from lithosphere to biosphere, sustaining large amounts of biomass, even beyond the hydrothermal fields [14–17]. As summarized in several reviews, the application of high-throughput sequencing technologies suggested that microorganisms in deep-sea hydrothermal ecosystems utilized all known biological carbon fixation pathways, including the Calvin-Benson (CBB) cycle, the reductive or reverse tricarboxylic acid (rTCA) cycle, the acetyl CoA pathway, the 3-hydroxypropionate bicycle, the dicarboxylate/4-hydroxybutyrate cycle, and the 3-hydroxypropionate/4-hydroxybutyrate cycle [18–20]. Also, these microorganisms have been proven to harness energy by oxidizing chemically reduced compounds from vent fluids, such as sulfide, hydrogen, methane, and metal ions [21]. Hydrothermal fluids are enriched with reduced sulfur compounds, which are identified as a predominant energy source for hydrothermal systems [11]. Sulfide-oxidizing aerobes are indicated to be the key primary producers in deep-sea hydrothermal systems [22]. The genera *Sulfurovum* and *Sulfurimonas* within the class Epsilonproteobacteria and some gammaproteobacterial members are thought to be the major sulfide-oxidizing autotrophs [9, 20, 22, 23]. Genomic analyses of these bacteria suggested that they used two sulfide-oxidizing pathways, the sulfur oxidation complex (SOX) pathway and the reverse sulfate reduction pathway to obtain energy for carbon fixation and other necessary metabolic processes [20, 22], by utilizing oxygen or nitrate as electron acceptors [20, 24, 25]. In addition, some studies have shown that chemoautotrophs in deep-sea hydrothermal vents can derive energy from hydrogen oxidation with oxygen [12, 20, 25, 26]. Thermodynamic models even estimate that hydrogen may provide more energy than sulfide in hydrogen-rich fluids [24, 27], indicating a significant role of hydrogen in the deep-sea hydrothermal ecosystems. Also, methane and iron are two available energy sources for chemoautotrophs, with representative utilizers including aerobic methanotrophs (Methylococcaceae and Methylocystaceae in the classes Gammaproteobacteria and Alphaproteobacteria, respectively), anaerobic methanotrophs (ANME-1 group), and iron oxidizers (*Mariprofundus ferrooxydan* in the class Zetaproteobacteria) [28–30]. Although previous research provided glimpses into the metabolic potentials of bacteria in deep-sea hydrothermal systems, investigation of the metabolic network of the whole microbial community in hydrothermal fields is lacking, limiting our understanding of the microbial element cycle in the deep-sea hydrothermal fields.

By the end of 2019, 718 hydrothermal vent fields had been identified along mid-ocean ridges (MORs), volcanic arcs, and back-arc spreading systems. More than half of them are located on MORs [31] (based on the InterRidge Vents Database, retrieved in December 2019 [1]). However, many MORs on Earth have rarely been explored for hydrothermalism [1]. For example, the MOR in the Atlantic Ocean south of 14°S remained unexplored until Cruise DY115-22 in 2011 [32] and Cruise MSM-25 of RV Maria S. Merian in 2013 [33]. During these cruises, 14 new hydrothermal vent fields were discovered [34], among which the 15.2°S hydrothermal field, also named the Deyin-1 hydrothermal field, has been the best characterized. This field was first discovered in 2011 [35], and its mineralogy and geochemistry were reported in 2017 [36]. In the Deyin-1 hydrothermal field, a number of violently erupting black smokers grow on a pillow-shaped basalt covered by a small amount of sediment, indicating that the oceanic crust is newly formed. Massive sulfide mineralization suggests that hydrothermal fluids mix with a significant amount of seawater during sulfide precipitation [36, 37]. In addition, the fungal, faunal, and ammonia oxidizer communities in this area were primarily investigated by analyzing marker genes [38–40], followed by the characterization of a new archaeal phylum (Hydrothermarchaeota) [41]. Collectively, these findings indicate a possible diverse microbial community in this field. However, little is known regarding the metabolic characteristics, element and energy transformations, and ecological adaptation strategies of the microbial community in the Deyin-1 hydrothermal field. Therefore, in the current study, metagenomes of four different hydrothermal samples were sequenced and assembled. Genomic bins were reconstructed, and the biodiversity and potential physiological capabilities of the microbial communities were characterized. The results shed light on energy capture, carbon and nitrogen acquisition, and metabolic interconnectivity in the deep-sea hydrothermal fields of this relatively unexplored area.

## Results

### Diverse microbial community in the Deyin-1 hydrothermal field

The sampling process has been described in previous mineralogical studies [36, 39]. In brief, four samples were obtained in the Deyin-1 hydrothermal field, including one sample composed of fragments of an active black smoker chimney (active chimney; TVG11), one sample with red-brown oxides obtained near an inactive chimney (hydrothermal oxide; TVG13), and two samples dredged from different inactive black smokers (inactive chimney; TVG10 and TVG12) [36, 39] (Figure S1 & Table S1). Differences in chemical parameters were observed among them, even between the two inactive chimney samples. For example, pH values of TVG10 and TVG13 were neutral, while those of TVG11 and TVG12 were more acidic; the total carbon and hydrogen content of TVG12 were lower than the other samples; and the total sulfur content of TVG13 was the lowest.

Metagenomic libraries of all four samples were constructed and sequenced, yielding more than 333 Gbp of DNA reads. After trimming, dereplication, and assembling, more than 12 Gbp scaffolds were assembled and subsequently clustered into MAGs (the details of the samples, metagenomes, and assembly are provided in Table S1). Finally, 615 constructed MAGs were estimated to be >25% complete and <10% contaminated. Their average completeness was 57.0%, and the average contamination was 3.5% (Table S2a). To enhance the reliability of this study, only 219 MAGs with estimated quality ≥ 50% (calculate as “completeness − 5 × contamination” [42]) were considered in the subsequent analyses. The average completeness of these 219 MAGs was 81.9%, and their average contamination was 2.9% (Table S2b).

**Fig. 1:**
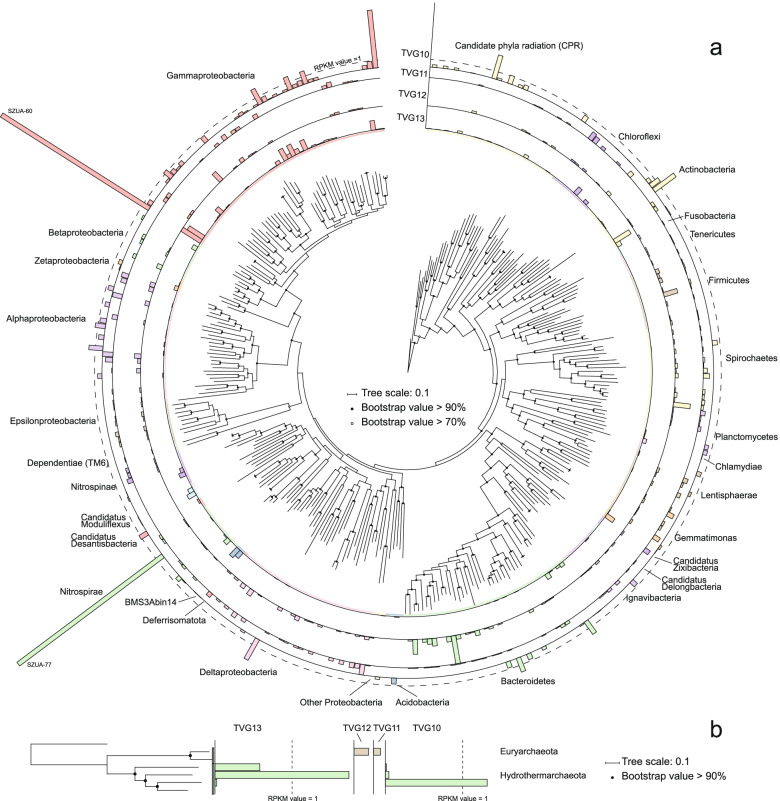
Phylogenetic tree and relative abundances of bacterial (**a**) and archaeal (**b**) MAGs in the current study. The maximum likelihood phylogenetic tree was constructed based on 120 bacterial and 122 archaeal marker genes. The scale bar represents 0.1 amino acid substitutions per sequence position. The bar plots are based on the relative abundance of each MAG in each sample, and the color of the bars represents the taxonomy of the MAG. The two most abundant MAGs (SZUA-60 and SZUA-77) are labeled. The full phylogenetic tree is available in Supplementary Data 1 (bacterial) and 2 (archaeal), and the details of MAG abundance are available in Table S2

**Fig. 2:**
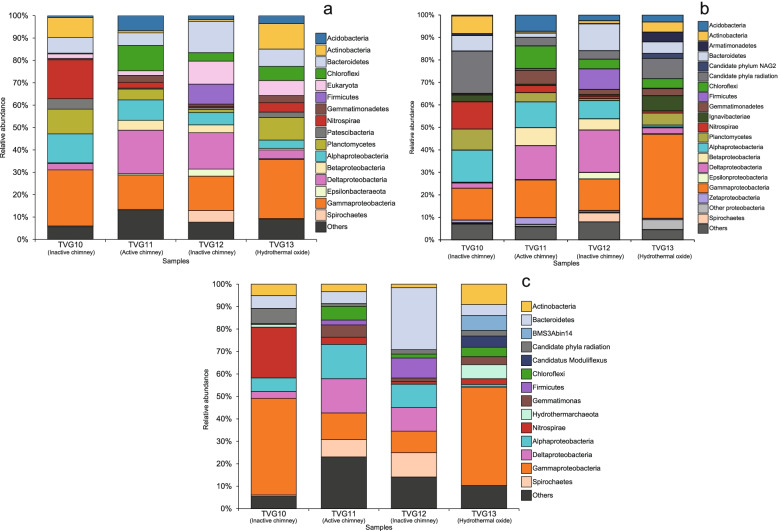
Microbial composition of each sample based on 16S rRNA genes (**a**), ribosomal protein S3 (**b**) and MAGs (**c**). The taxonomy of 16S rRNA genes was determined by comparing with SILVA database; the taxonomy of ribosomal protein S3 was determined by comparing with NR database; the taxonomy of MAGs was determined by considering the results of 16S rRNA genes and genomic phylogeny

**Fig. 3:**
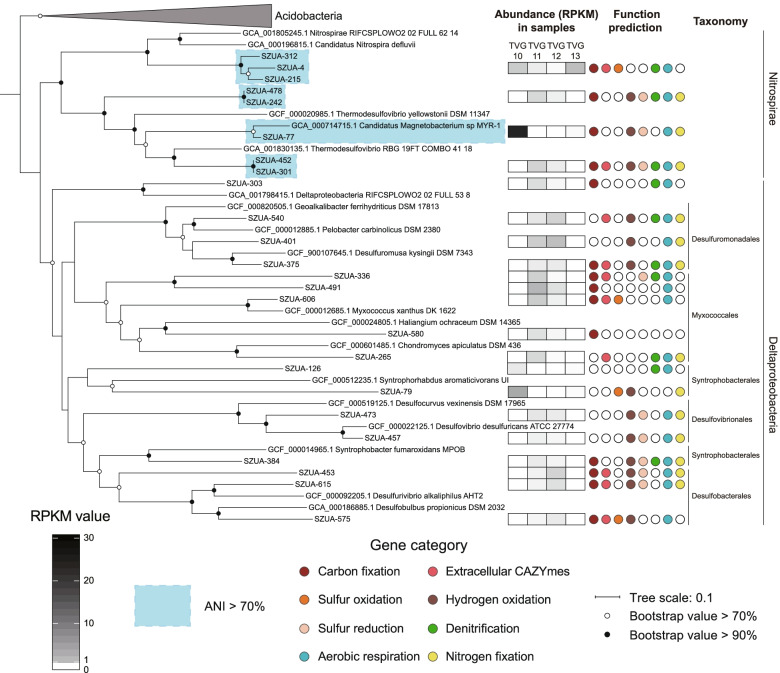
Phylogenetic tree, relative abundance, and functional potentials of Nitrospirae and Deltaproteobacteria MAGs. The maximum likelihood phylogenetic tree was constructed based on 120 bacterial marker genes. The scale bar represents 0.1 amino acid substitutions per sequence position. RPKM value is the relative abundance of each MAG, calculated by number of mapped reads/(sequence length × metagenomic size)

To assess the taxonomic composition of the communities in these samples, the taxonomic abundance of 16S rRNA gene (16S), ribosomal protein S3 (rpS3), and MAGs were considered. In total, 1743 16S sequences (length >700 bp) and 3291 rpS3 sequences were obtained from the scaffolds. All 16S rRNA gene sequences were clustered into operational taxonomic units (OTUs) with 95% similarity cutoff, and the rpS3 sequences were clustered with 70% similarity. The phylogenies of these two marker genes were conducted with representative sequences of the OTUs, respectively. The phylogeny of the 16S rRNA genes showed that these sequences were assigned to 52 phyla (45 Bacteria and 7 Archaea). Most of 16S sequences were bacterial sequences (1631), and the three most abundant sequences all belonged to Gammaproteobacteria (Figure S2). The taxonomic assignments of rpS3 sequences showed that at least 85 phyla (76 Bacteria and 9 Archaea) exist in these samples. Also, most of rpS3 sequences belonged to Bacteria (3220). The phylogeny of the MAGs showed the same pattern, with 219 MAGs assigned to 26 phyla (24 Bacteria and 2 Archaea), where 215 MAGs belonged to Bacteria (Fig. 1 & S3). The abundance of each MAG was calculated with the mapped reads and normalized using “Reads per kilobase per million mapped reads” (RPKM) method [43]. The most abundant MAG (SZUA-60) was also affiliated with Gammaproteobacteria (RPKM value of 6.9), followed by SZUA-77, a member of the phylum Nitrospirae (RPKM value of 6.3) (Fig. 1). A summary of the taxonomic abundances based on 16S rRNA gene, rpS3, and MAGs is shown in Fig. 2–c. All these results reached an agreement that the abundance of Bacteria was much greater than that of Archaea (2.3, 3.3, and 2.0% for 16S, rpS3, and MAGs). Gammaproteobacteria (20.6, 20.6, and 18.8% for 16S, rpS3, and MAGs) was the most abundant taxon in the Deyin-1 hydrothermal field, followed by Delta-, Alphaproteobacteria, Bacteroidetes, and Nitrospirae (10.6, 7.8, 8.6, and 6.2% for 16S; 9.7, 8.7, 6.5, and 4.4% for rpS3; 7.3, 8.2, 10.9, and 7.4% for MAGs). It should be mentioned that Candidate Phyla Radiation (CPR) was highly abundant in the results of rpS3 (8.9%), but it was not detected in the results of 16S rRNA genes. This difference may be caused by the limitation of metagenome assembly for 16S rRNA genes, as the sequences of 16S rRNA gene are highly conserved [44]. Also, the intron inserted in the 16S rRNA genes of CPR may be another barrier for assembling them [45]. To further elucidate the taxonomic position of the MAGs within these five major taxa, phylogenetic analyses were performed, indicating a great diversity within these taxa (Figs. 3, S4, S5, & S6). Notably, the two most abundant MAGs, SZUA-60, and SZUA-77, phylogenetically belonged to *Acidiferrobacter* and *Candidatus* Magnetobacterium (within the family Nitrospiraceae), respectively (Figs. 3 & S5). Both groups were also the two most abundant genera in the Deyin-1 hydrothermal field (10.2 and 5.5% for MAGs, respectively).

From a taxonomic perspective, marked differences in the microbial community composition were observed across samples, even between two inactive chimney samples. For instance, Gammaproteobacteria was the most abundant taxon in samples TVG10 and TVG13 (25.1 and 26.5% for 16S, 14.2 and 37.4% for rpS3, and 43.0 and 43.8% for MAGs), while Deltaproteobacteria was the most abundant taxon in samples TVG11 and TVG12 (19.3 and 16.3% for 16S, 15.1 and 18.9% for rpS3, and 15.3 and 10.6% for MAGs). In addition, the relative abundances of some phyla were greater than 5% in only one of the four samples (called “abundant” below). Specifically, Nitrospirae was only abundant in TVG10 (17.5, 12.2, and 22.6% for 16S, rpS3, and MAGs). Chloroflexi and Gemmatimonas were only abundant in TVG11 (11.3, 10.1, and 22.6%; 3.1, 6.2, and 5.5% for 16S, rpS3, and MAGs). And Firmicutes was only abundant in TVG12 (8.9, 9.2, and 8.9% for 16S, rpS3, and MAGs) (Fig. 2). Besides, the Venn diagram of the 16S rRNA gene OTUs and multidimensional scaling (MDS) analysis of the taxonomic abundance showed the distinction of these four microbial communities. According to the Venn diagram, only 1.5% of OTUs were shared by all samples. The shared OTUs between two inactive chimney samples accounted for merely 3.4%. More than half of the OTUs were exclusive to specific samples (Figure S7a). The MDS results revealed that the distance between two inactive chimney samples TVG10 and TVG12 was larger than that between TVG12 and active chimney sample TVG11, suggesting great distinctions of the microbial composition among these samples (Figure S7b). Meanwhile, the rarefaction curves showed that the observed OTUs of 16S rRNA gene in samples TVG10, TVG11, and TVG13 plateaued at 10000-sequence level (Figure S8a). Similarly, for the sample TVG12, the rarefaction curve of chao1 index plateaued at 10000-sequence level (Figure S8b), indicating adequate sampling of major prokaryotic communities for all samples. With these considered, the substantially distinct microbial communities across these samples are possibly shaped by fluctuating environmental conditions in the hydrothermal systems, such as pH, total hydrogen, and total sulfur.

### Energy acquisition and electron acceptors

All the MAGs were annotated and analyzed for genes involved in energy acquisition and electron-accepting pathways. Several pathways were predicted in all four samples, including those for sulfur, hydrogen, nitrogen, and aerobic metabolisms (Table S3).

**Fig. 4:**
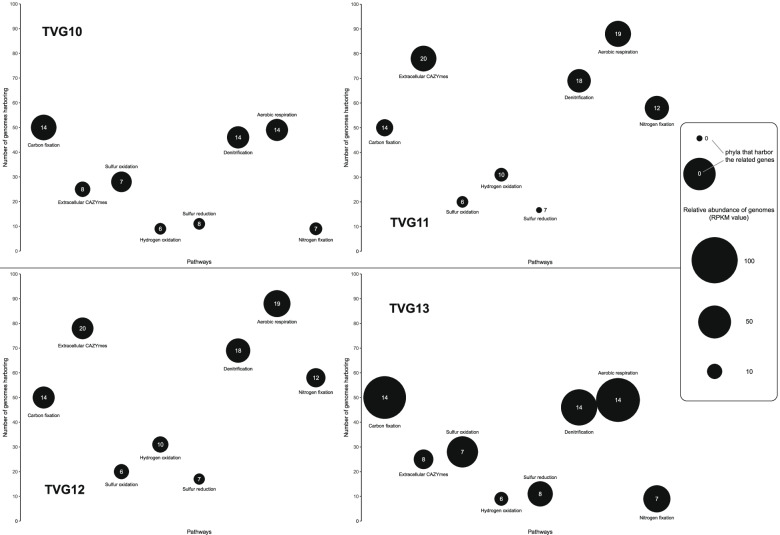
Relative abundance of MAGs, numbers of MAGs, and phyla involved in different pathways in each sample. The numbers in the circle represent the number of phyla harboring the related genes

As for the metabolic pathways related to energy acquisition, many MAGs had both sulfur and hydrogen oxidation capabilities. In the samples TVG10 and 13, the number of MAGs containing genes of sulfur oxidation was higher than the number of those harboring genes of hydrogen oxidation, while the results were opposite in TVG11 and 12 (Fig. 4). Thus, both metabolisms were important energy sources in the hydrothermal fields. However, the relative abundance of MAGs with the potential for sulfur oxidation (RPKM value of 19.8) was higher than that for hydrogen oxidation (RPKM values of 8.3; Fig. 4). The difference in these gene abundances suggested that sulfur oxidation might be the major energy source and hydrogen oxidation might be a minor source in the hydrothermal fields. It should be noted that this inference from metagenomic study needs to be further verified by gene activities. Taxonomically, the orders Rhizobiales and Rhodobacterales within Alphaproteobacteria together with gammaproteobacterial members may be the major microbial groups for oxidizing sulfur in the hydrothermal field, reflected by the sulfur-oxidizing genes (including *rdsrAB*-*aprAB*-*sat* and SOX complex) detected in a high abundance of their MAGs in all four samples (Figure S9) (RPKM values of 0.6, 1.5, and 14.7 for Rhizobiales, Rhodobacterales, and Gammaproteobacteria, respectively; Figures S4 & S5). On the other hand, Nitrospirae and Deltaproteobacteria were likely the major microbial groups for oxidizing hydrogen, as the relative abundance of their MAGs harboring genes related to this process were 6.7 and 0.8 (RPKM value), respectively (Fig. 3). In addition, the genes for methane production (*mcrABC*) were only observed in euryarchaeotal MAG SZUA-459 with low abundance (Table S3), and those for methane oxidation were only observed in a low-quality gammaproteobacterial MAG SZUA-363, indicating that methane should be a less important energy source for the microbial community in the Deyin-1 hydrothermal field.

Furthermore, the high abundance of genes related to sulfur reduction, denitrification, and aerobic respiration suggested that oxidized sulfur, nitrite, and oxygen may be the most common electron acceptors for microbial community in the Deyin-1 hydrothermal field. Among these pathways, denitrification and aerobic respiration were the most widespread, and the relative abundance of MAGs harboring the key genes involved in these two pathways were 33.7 and 44.0 (RPKM value), respectively (Fig. 4). With respect to aerobic respiration, cbb3-type terminal cytochrome c oxidase (*ccoNOP*) was the most common genes (present in the MAGs with RPKM values of 15.8–60.7), followed by cytochrome d oxidase (*cydAB*) (present in the MAGs with RPKM values of 0.8–26.5). In comparison, the MAGs with relative abundance of only 0.6–2.8 (RPKM value) had cytochrome o (*cyoABCDE*), and cytochrome c oxidase (*coxABC*) was only found in members of Bacteroidetes and Deltaproteobacteria (Table S3). In terms of sulfate reduction, *Candidatus* Magnetobacterium within the phylum Nitrospirae, abundantly present in TVG10, contained a large number of related genes for this process (Fig. 3), suggesting that Nitrospirae may be a major sulfate reducer in TVG10. In contrast, in samples TVG11 and TVG12, the abundance of sulfate reducers within the class Deltaproteobacteria, including Desulfovibrionales, Desulfobacterales, and Syntrophobacterales, was high rather than those within Nitrospirae (Fig. 3). Thus, unlike TVG10, deltaproteobacterial members are likely the major sulfate reducing taxa in TVG11 and TVG12. In addition, the gene of polysulfide reductase (*psrA*) was found in the Nitrospirae, Delta-, Gamma-, and Zetaproteobacteria MAGs. The *psrA*-containing MAGs within Nitrospirae and Deltaproteobacteria were only found in TVG11 and TVG12, while those within Gamma-, and Zetaproteobacteria were only present in the other two samples, suggesting significant contribution of these taxa to sulfur reduction in the hydrothermal fields (Table S3).

Notably, among the diverse taxa detected in the Deyin-1 hydrothermal field, Gammaproteobacteria MAGs contained abundant genes related to all mentioned energy-producing pathways (sulfur/sulfide, hydrogen, and methane oxidation; Figure S5), which was in agreement with their copiotrophic lifestyle represented in the previous studies [46, 47]. Thus, Gammaproteobacteria potentially plays an important role in organic carbon production in the Deyin-1 hydrothermal ecosystem. Besides, sulfur and hydrogen oxidation pathways were found in *Acidiferrobacter* and Thiotrichales, enabling their energy production through these compounds. In contrast, a few mentioned metabolic pathways were only detected in MAGs of other lineages in specific samples. For example, Alphaproteobacteria potentially utilized diverse chemical compounds except for hydrogen (Figure S4), and Bacteroidetes possessed abundant genes associated with hydrogen oxidation in TVG12 (Figure S9 & Table S3).

### Carbon fixation and extracellular carbohydrate-active enzymes (CAZymes)

To predict potential metabolic pathways for carbon cycling, the abundances of genes encoding key enzymes for carbon fixation and CAZymes were calculated, particularly for the CAZymes predicted to be secreted (extracellular CAZymes). The results indicated that key genes associated with carbon fixation were present in all four samples in abundance. Specifically, diverse carbon fixation pathways were predicted in the MAGs with RPKM values of 10.0–55.4 (Fig. 4), including the CBB cycle, the rTCA cycle, the Wood-Ljungdahl pathway, the 3-hydroxypropionic acid pathway, and the 4-hydroxybutyryl pathway (Figure S9). Nitrospirae, Alpha-, Delta-, and Gammaproteobacteria were the most abundant taxa involved in carbon fixation, as the relative abundance of their MAGs harboring the key genes were 7.2, 2.8, 1.8, and 8.2 (RPKM value), respectively. In particular, Nitrospirae and Gammaproteobacteria were abundant carbon-fixing taxa across all four samples (Figure S9).

In addition, 18,622 genes encoding potential CAZymes were detected in four samples, and approximately 2.7–5.2% of them were predicted to be secreted (Figure S10). Among these extracellular CAZymes, GH13 (α-amylase), GH15 (glucoamylase), GH16 (active toward β-1,4 or β-1,3 glycosidic bonds), GH57 (α-amylase), GH74 (xyloglucanase), and PL22 (oligogalacturonate/oligogalacturonide lyase) were detected in all samples (Table S4). The relative abundance of MAGs encoding extracellular CAZymes in the samples (RPKM values of 14.4–31.4) was comparable with that having carbon-fixing genes, suggesting that extracellular carbohydrate compounds could be another important carbon source for these sampled microbial communities in the Deyin-1 hydrothermal field. Contrastingly, for the hydrothermal oxide sample (TVG13), the number of MAGs encoding extracellular CAZymes was much lower than that possessing carbon fixation pathways (Fig. 4), suggesting that carbon fixation might be more prevalent in this hydrothermal oxide sediment sample. Of note, co-occurrence of carbon fixation and extracellular carbohydrate utilization pathways was observed in 41 MAGs (RPKM values of 5.8–9.4; 32 of them were with contamination <5%), including the MAGs within known chemoautotrophic taxa (*Acidiferrobacter*, Thiotrichales, etc.). Altogether, these results suggest that these autotrophic members exhibit potentials to use both heterotrophic and autotrophic strategies for survival in deep-sea hydrothermal ecosystems.

### Nitrogen fixation

**Fig. 5:**
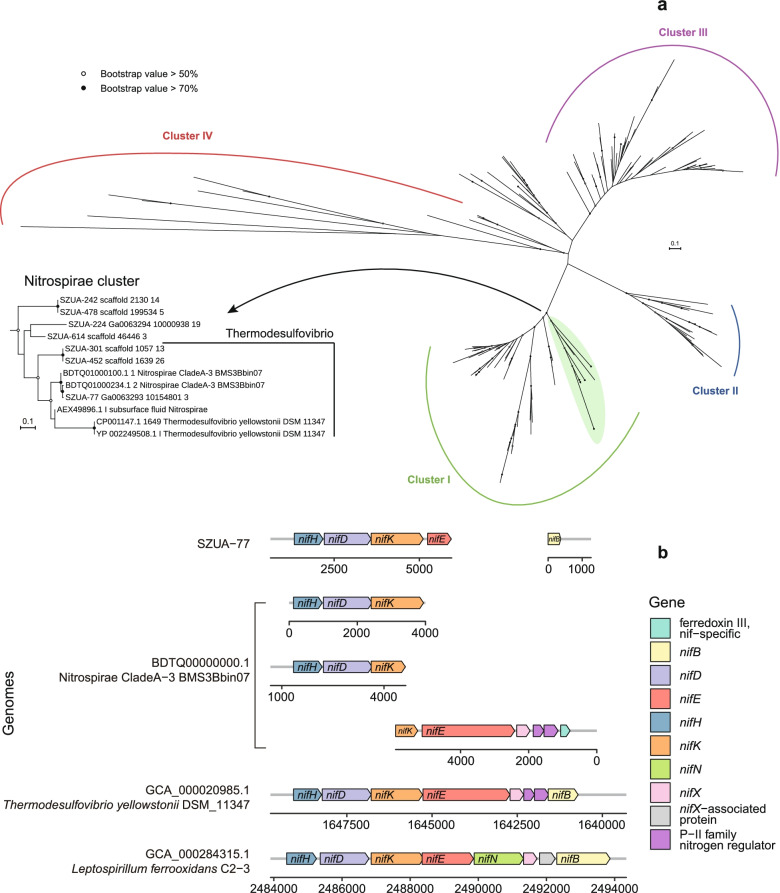
Phylogenetic tree of NifH sequences from MAGs (**a**) and comparison of the *nif* operon from SZUA-77, the closest *nif* operon in NCBI, and the *nif* operons of representative Nitrospirae diazotrophs (**b**). The scale bar in the phylogenetic trees represents 0.1 amino acid substitutions per sequence position. The information of reference NifH sequences used in the phylogeny is in Table S6

The key genes of nitrogen fixation (*nifHDK*) were observed in the inactive chimney samples, with the RPKM values as high as 207.7 in TVG10 and 165.3 in TVG12 (Table S3). Relevant genes were found in 61 MAGs within diverse taxa, including Bacteroidia (within Bacteroidetes), Nitrospirae, Rhodospirillaceae (within Alphaproteobacteria), Deltaproteobacteria, and Thiotrichales (within Gammaproteobacteria) (Fig. 3, Figs. S4, S5, & S6). In particular, a cascade of genes related to nitrogen fixation was present in the MAG SZUA-77 (*nifHDKEB* in Fig. 5b), which was one of the most abundant MAGs and phylogenetically belonged to *Candidatus* Magnetobacterium (Fig. 3), suggesting its potential nitrogen fixation ability. Highly abundant and diverse nitrogen-fixing communities imply that substantial nitrogen fixation possibly takes place at deep-sea hydrothermal sites, and dinitrogen may be an essential nitrogen source for the microbial community in situ.

## Discussion

As a representative of slow-spreading MORs, the Mid-Atlantic Ridge is estimated to have a spreading rate of ~35 mm/year [48], attracting considerable research interest in recent years [6, 41, 49–52]. However, the Mid-Atlantic Ridge south of 14°S remained unexplored until recent cruises in which 14 hydrothermal deposits were confirmed [32]. Among them, the Deyin-1 hydrothermal field is a Normal-Mid-Ocean Ridge Basalt-hosted field with a locally confined hydrothermal plume [34, 37]. Hydrothermal fields have long been characterized by intensive fluctuations in salinity and temperature. The salinity ranges from 0.1 to twice the salinity of seawater [53], and the temperature ranges from 2°C when mixing with seawater to more than 400°C at the core vent [54]. It was observed in a previous study that the pillow-shaped basalt was covered by small volume of sediment in the Deyin-1 hydrothermal field, indicating that the oceanic crust in this field was newly formed [36]. Hydrothermal activity at newly formed fields is usually unstable, it could lead to more fluctuating environments in situ, making Deyin-1 hydrothermal field an ideal site to study the hydrothermal influence on the microbial community in young hydrothermal vents [36, 37]. In the current study, 333.3 Gbp of metagenomic data were obtained, which unveiled highly diverse and variable microbial communities in the Deyin-1 hydrothermal field, including 219 high-quality MAGs within 26 phyla (Fig. 1). Compared to the microbial compositions of the deep seafloor sediments in Atlantic Ocean [55–57], some microbial groups were exclusively found in our samples, such as Nitrospirae and Hydrothermarchaeota. These taxa have been widely detected in hydrothermal fluid samples [55–57], suggesting a noticeable influence of the hydrothermal fluid activity on microbial communities. This study is the first to investigate the microbial compositions, energy acquisition and electron-accepting pathways, as well as carbon and nitrogen metabolic pathways in the hydrothermal ecosystem at the Mid-Atlantic Ridge south of 14°S. The results of microbial diversity and metabolisms in this newly discovered hydrothermal field provide another example of metabolic plasticity and functional redundancy for the microbial community in deep-sea hydrothermal systems.

### Variable microbial communities and their energy sources

In the current study, Bacteroidetes, Nitrospirae, Alpha-, Delta-, and Gammaproteobacteria were the five most abundant taxa. While only Alpha- and Gammaproteobacteria represented major constituents in sampled microbial communities, the abundance of Deltaproteobacteria and Nitrospirae exhibited great variations, even between two inactive chimney samples (Fig. 2). The taxonomic variation was also observed in the results of both MDS analysis and Venn diagram (Figure S7). A possible reason is the highly fluctuating or varying environments in situ. As for the energy sources, consistent with the previous hydrothermal studies [21], sulfide and hydrogen might be two vital energy sources for the microbial ecosystem in the Deyin-1 hydrothermal field. Sulfide could be the major energy source for Alpha- and Gammaproteobacteria, while hydrogen might provide energy mainly for Nitrospirae and Deltaproteobacteria (Fig. 3, Figs. S4 & S5).

In agreement with the previous studies [49, 58], Nitrospirae was one of the major bacterial phyla in the inactive chimney sample TVG10 (Fig. 2). However, it showed lower abundance in the other three samples, including the inactive chimney sample TVG12 (Fig. 2). It is possible that a much lower pH value in TVG12 may affect the growth of Nitrospirae. Additionally, both Nitrospirae and Deltaproteobacteria were predicted to be the major hydrogen oxidizers and sulfate reducers. Nitrospirae was only abundant in TVG10, while Deltaproteobacteria was abundant in TVG11 and TVG12, where the environments were more acidic than TVG10 (Fig. 3). Deltaproteobacteria is one of the most common sulfate-reducing taxa in some acidic environments (such as acid mine drainage) [59, 60], and some acid-tolerant deltaproteobacterial members were cultured from acid mine drainage [61–63]. Thus, the relatively low pH value of the samples TVG11 and TVG12 seemed to be one of the important reasons for the taxonomic shift of hydrogen oxidizers, rather than different temperatures between active and inactive chimneys (TVG 11 and TVG12).

Inconsistent with previous studies showing the overwhelming predominance of Epsilonproteobacteria in active chimney rocks from Manus Basin and Southern Mariana Trough [49, 64], this class only occupied less than 2% of total prokaryotes in the active chimney sample TVG11. It is believed that Epsilonproteobacteria are sulfur oxidizers dependent on reduced sulfur from fluids in active chimneys [49]. Their low abundance detected in our study indicated that the fluids from the active chimney TVG11 might not be rich in reduced sulfur. In addition, a previous study reported that thermophilic archaea (Bathyarchaeota and Euryarchaeota) were dominant in hydrothermal sediments from Guaymas Basin, where organic carbon was plentiful [51]. But they were less abundant in our samples. Since these archaeal groups are known for utilizing extracellular organic carbon [7, 65], an explanation is that the samples taken from Deyin-1 hydrothermal field are not rich in organic carbon.

Moreover, methane has been reported to be one of the crucial energy sources for the hydrothermal ecosystems in Guaymas Basin, Menez Gwen, and Rainbow vent fields, which might be attributed to the sediment rich in organic carbon or the fluids with high methane concentrations [51, 52]. In the current study, methanotrophic pathways were only detected in one MAG with less abundance (Table S3), suggesting a possible low concentration of methane in the hydrothermal fluids and a minor role of methane metabolisms in the Deyin-1 hydrothermal field.

*Co-existence of heterotrophic and autotrophic pathways*

Regarding microbial carbon metabolism, except for Bacteroidetes, high relative abundance of MAGs within the other four major taxa (RPKM values of 1.8–8.2) had the potential to fix inorganic carbon (Table S3). This result suggested a high proportion of carbon-fixing microbes in the Deyin-1 hydrothermal field, in agreement with the results of previous studies on other hydrothermal sites [49, 50]. Similar to the results of a previous study on the Guaymas Basin hydrothermal plume [6], genes encoding CAZymes were frequently detected in the MAGs, with a high proportion of extracellular CAZymes (Figure S10). For the samples TVG11 and TVG12, the numbers of genomes encoding extracellular CAZymes were even higher than those containing carbon-fixing genes (Fig. 4), suggesting an important role of heterotrophic pathways for the hydrothermal ecosystem, especially for active chimneys. Notably, the genes of extracellular CAZymes co-occurred with carbon fixation pathways in many MAGs with contamination < 5% (Tables S3 & S4), and their abundance was in accordance with that of carbon fixation genes in diverse taxa (Figures S9 & S10), indicating that both carbon fixation and extracellular carbohydrate utilization pathways were potentially essential for microorganisms inhabiting deep-sea hydrothermal ecosystems. In these extreme environments, particularly in active chimney sites, high carbon fixation productivity supports large amounts of biomass, which could explain the high abundance of genes related to heterotrophic metabolisms. In the hydrothermal field, sharply fluctuating environments may kill many organisms and produce enormous amounts of organic detrital compounds, enriching heterotrophic microbes and even encouraging some chemoautotrophic members to utilize surrounding carbohydrates. Thus, extracellular carbohydrate utilization pathways should be indispensable for carbon recycling in deep-sea hydrothermal ecosystems. Moreover, the co-occurrence of carbon fixation and extracellular carbohydrate utilization pathways in many MAGs indicates the presence of redundant carbon-obtaining capabilities for many microbes, which may be their survival strategy in the extreme hydrothermal environment.

### Novel nitrogen-fixing bacteria

Dissolved dinitrogen gas is one of the largest nitrogen reservoirs in the ocean and presents in large amounts in hydrothermal fluids [66, 67]. Biological nitrogen fixation has been suggested as an important nitrogen source for deep-sea hydrothermal ecosystems [68]. Although multiple methods (including culture-, amplification-, and metagenomic-based) have been used to predict the nitrogen fixation capabilities of microorganisms in hydrothermal ecosystems [67, 69–71], our understanding regarding the community structure and metabolism of diazotrophs in this environment is limiting.

The observation in the current study was that nitrogen fixation represented a widespread and abundant pathway for multiple taxa across all samples (Fig. 4, Fig. S9 & Table S3). Also, phylogenetically diverse *nifH* sequences were detected in the metagenomes (Fig. 5a). Taking into consideration the predicted metabolic profiles of these MAGs, a wide range of chemicals in hydrothermal environments (including carbohydrates, sulfide/sulfur, hydrogen) could provide energy for nitrogen fixation. Thus, in agreement with the results of a previous study of other hydrothermal ecosystems, dinitrogen should be a major nitrogen source for the microbial community in different types of samples at the Deyin-1 hydrothermal field [72].

According to the previous studies of the nitrogenase gene *nifH*, methanogenic archaea, Firmicutes, Nitrospirae, and Proteobacteria were considered the potential nitrogen fixers in deep-sea hydrothermal vents [67, 72]. However, in the current study, in addition to those typical proteobacterial diazotrophs (Nitrospirae, Delta-, and Gammaproteobacteria), abundant *nifHDK* genes were observed in the MAGs of the phyla Spirochaetes and Bacteroidetes (class Bacteroidia) from the sample TVG12 (Fig. S6, Fig. S9), indicating their nitrogen-fixing potential in the inactive chimney. Members of the phylum Spirochaetes are important diazotrophs that have only been detected in insect guts and freshwater environments [73, 74]. In comparison, diazotrophs within Bacteroidia inhabit diverse environments, including the seafloor [75, 76], but have not been reported in deep-sea hydrothermal ecosystems. This study is the first to report the potentially nitrogen-fixing Bacteroidetes and Spirochaetes inhabiting the inactive hydrothermal chimney, suggesting that some members within these two phyla may play important roles in the nitrogen cycle.

In addition, the *nif* operon, including the genes *nifHDK*, was present in the MAG SZUA-77, which was one of the most abundant MAGs in the inactive chimney sample TVG10 (Fig. 3). Phylogenetic analysis of NifH sequences showed that the sequences present in SZUA-77 clustered with the reference Nitrospirae sequences within group I (Fig. 5a). The results of comparative analyses of gene clusters also indicated that the cluster structure of SZUA-77 was highly syntenous with that of Nitrospirae BMS3Bbin07 and the nitrogen fixer *Leptospirillum ferrooxidans* C2-3 [77, 78] (Fig. 5b). Collectively, these findings reveal a great potential of nitrogen fixation by SZUA-77. On the other hand, phylogenetic analyses of Nitrospirae genomes showed that SZUA-77 clustered with the MAGs of *Candidatus* Magnetobacterium (Figure S11a). The percentages of conserved proteins between SZUA-77 and the MAGs of *Candidatus* Magnetobacterium were higher than 50% (50.2–58.4%; Figure S11b), which was a proposed genus boundary for prokaryotes [79]. Accordingly, the MAG SZUA-77 should be affiliated with the genus *Candidatus* Magnetobacterium. It is generally known that members of this genus are widespread in diverse habitats, including aquatic environments [80], estuaries [81], seafloor [82], and hydrothermal fields [83], with unique characteristics of forming bullet-shaped magnetite magnetosomes and arranging multiple magnetosome chains [84, 85]. In agreement with previous genomic analyses on *Candidatus* Magnetobacterium, potential pathways for carbon fixation, sulfate reduction, denitrification, aerobic respiration, and hydrogen oxidation (NiFe hydrogenase group 3b) were present in SZUA-77 [84] (Fig. 3). Nevertheless, no genes related to magnetosome biomineralization were found. Instead of utilizing nitrate or nitrite as nitrogen source described previously [84], the high similarity of both *nifHDK* sequences (94.6–99.3%) and operon structure suggested the potential diazotrophic ability of SZUA-77 within *Candidatus* Magnetobacterium. Considering *Candidatus* Magnetobacterium showed a high abundance in the sample TVG10 but was barely found in the more acidic sample TVG12, it could be inferred that *Candidatus* Magnetobacterium likely fixes nitrogen in the inactive chimney under neutral pH conditions.

### Metabolic plasticity and functional redundancy

Metabolic plasticity refers to the potential to switch metabolic processes in response to changing environments [51], which was observed in the major members of the microbial community in this study. For example, Alpha- and Gammaproteobacteria could couple sulfur oxidation with either denitrification or aerobic respiration (Figures S4 & S5). Nitrospirae and Deltaproteobacteria could couple hydrogen oxidation with either sulfate reduction, denitrification, or aerobic respiration to obtain energy (Fig. 3). Furthermore, a large proportion of the major taxa were potentially capable of obtaining organic carbon by fixing inorganic carbon and utilizing extracellular carbohydrates. In fact, many studies have reported the metabolic plasticity of bacterial members in deep-sea hydrothermal ecosystems [51, 86, 87], suggesting that this might be a common feature for the hydrothermal microorganisms. A possible reason is that these additional metabolic pathways provide multiple surviving strategies, potentially allowing microbial adaptation to fluctuating deep-sea hydrothermal habitats.

As for the microbial community in situ, great distinctions in microbial composition were observed across the four samples, even between the two inactivate samples (Figure S7), which was similar to observations in other hydrothermal ecosystems [88, 89]. Diverse microbial communities in deep-sea hydrothermal fields could serve as flexible seed banks that allow the communities to survive in the highly fluctuating environments in the ground [90, 91], suggesting that the variation of community composition is an adaptation strategy for the community as a whole to persist in such extreme environments.

**Fig. 6:**
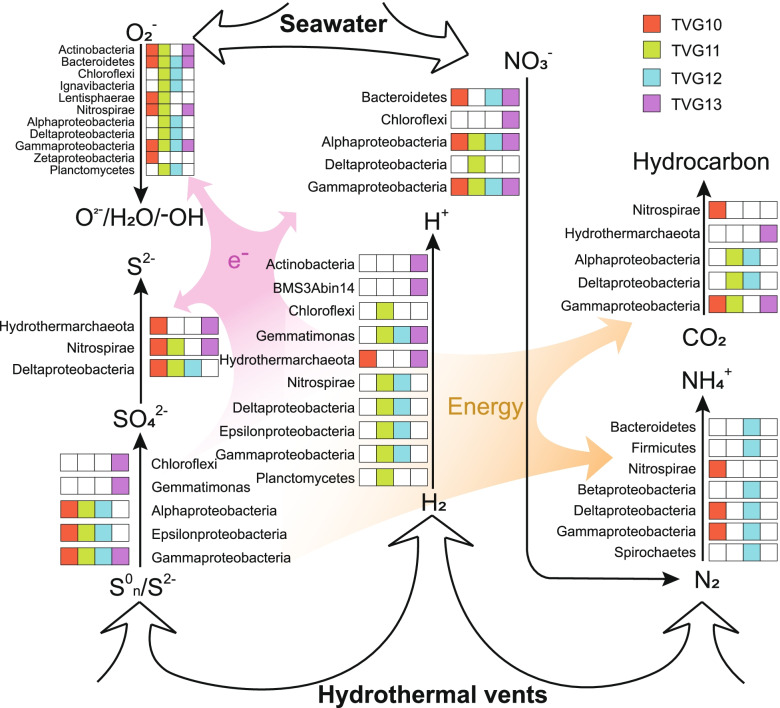
Metabolic network and the involved taxa in the hydrothermal ecosystem. The heatmaps show the presence of metabolic genes harbored by the taxa in each sample. The brown arrow shows the transfer of energy, and the purple arrow shows the transformation of electrons

From the perspective of community metabolism, taxonomically distinct microorganisms in different samples harbored the same metabolic pathways. For example, abundant sulfate-reducing Nitrospirae were present in TVG10, while Deltaproteobacteria was the major sulfate reducer in TVG11 and TVG12 (Fig. 3). Aggregating the MAGs potentially involved in carbon metabolism, nitrogen fixation, and energy acquisition led to the observation that at least three taxa could participate in each step (Fig. 6), suggesting a high degree of functional redundancy across the microbial communities in the Deyin-1 hydrothermal field. Reported in other hydrothermal systems [92, 93], the functional redundancy of the microbial community is considered to be the key to ensure the stability of metabolic processes despite the elimination of certain taxa by fluctuating environments [51, 94]. A recent study found that functional redundancy may be an inevitable emergent property as a consequence of mainly biotic interactions and environmental and spatial processes [95]. Another theory—“it’s the song not the singer” (ITSNTS)—assumes that the biochemical functions (“the song”) of the microbial community (“the singers”) are more conserved and ecologically relevant than the microbial community itself [96]. Specifically, the transformation of chemicals and energy within specific environments can be maintained by taxonomically diverse but functionally similar microorganisms. Instead of microbial compositions, collective metabolic functions of the microbial community are proposed as general characteristics for specific environments. Overall, in fluctuating environments like deep-sea hydrothermal fields, the metabolic plasticity of microbes, the high biodiversity of community compositions, and the functional redundancy of microbial communities may be common features for maintaining the stability of the metabolic network in situ.

## Conclusions

The Southern Mid-Atlantic Ridge represents a slow-spreading ocean ridge on Earth. So far, little is known about the deep-sea hydrothermal systems along this ridge. Deyin-1 is a newly discovered hydrothermal field in the Atlantic Ocean south of 14°S with diverse communities of fauna and microbes described [38–40], indicating it is an ideal site to study the microbial diversity and metabolisms of deep-sea hydrothermal system in the South Atlantic Ocean. In the current study, we first investigated the microbial composition and functional potential in this field via MAG construction, revealing a phylogenetically diverse and variable community, with Bacteroidetes, Nitrospirae, Alpha-, Delta-, and Gammaproteobacteria as the major taxa. In the metabolic aspect, reduced sulfur and hydrogen were the primary energy sources for microbial chemosynthesis, and frequent observation of nitrogen fixation pathways in the major taxa revealed dinitrogen as one of the major nitrogen sources. Of note, taxonomically different bacteria exhibited the same metabolic potentials across samples, suggesting a highly variable community possibly impacted by the fluctuating environments. The co-occurrence of carbon fixation and extracellular carbohydrate utilization pathways in most MAGs indicated a possible surviving strategy by using both heterotrophic and autotrophic pathways, which highlights the metabolic plasticity of the microorganisms in situ. Intriguingly, for the first time, the genus *Candidatus* Magnetobacterium was predicted to be a potential nitrogen fixer, and the diazotrophs within the phyla Bacteroidetes and Spirochaetes were detected in the inactive chimney. Moreover, the metabolic plasticity of microbes, diverse and variable community composition, and functional redundancy of microbial communities may be an adaption strategy to maintain the metabolic network in the geochemically complex and fluctuating environmental conditions in deep-sea hydrothermal fields.

## Methods

### Sample collection, DNA extraction, and sequencing

As described previously, the Deyin-1 hydrothermal field (15.2°S hydrothermal field of the Mid-Atlantic Ridge), located between the Cardno and St. Helena Fracture zones, was explored for hydrothermal activity during a cruise of the R/V *Dayangyihao* in August 2012 [37, 39] (Figure S1). One hydrothermal oxide, one active, and two inactive chimney samples were collected by a TV-guided grab sampler and stored at −80°C before subsequent DNA extraction. The chemical parameters, including total C, total N, total H, total S, and the C/N ratio, were analyzed using a Vario EL III elemental analyzer (Elementar, Germany). The pH value was determined in 1:1 sample/water slurries (Table S1). The pictures, chemical parameters, and more detail information of these samples could be found in the previous study [37].

All of the samples were moved from the container with sterile strips, placed on the sterile petri dishes, and rinsed with sterile seawater. Then, they were crushed with a sterile pestle and mortar, aliquoted to the same quantity, and placed into the DNA extraction tubes. Genomic DNA from all samples was extracted using a FastDNA® Spin kit for Soil (MP Biomedicals, USA) according to a modified extraction protocol [97]. Triplicate DNA extracts were pooled for each sample and stored at −20°C until use. The extracted DNA was examined by 1.0% agarose gel electrophoresis. The qualified DNA samples were randomly fragmented by sonication. The DNA fragments were end polished, A-tailed, and further PCR amplified (28 cycles). The PCR products were purified with AMPure XP system, sheared, and ligated with adapter sequences to prepare the DNA libraries. The libraries were then sequenced on an Illumina HiSeq 2000 (USA) PE 150 (paired-end reads of 2 × 150 bp) platform by Novogene Corporation (China).

### De novo metagenomic assembly and taxonomic analysis

Raw sequencing reads of all samples were dereplicated (100% identity over 100% length; dereplicate script from https://github.com/Geo-omics/scripts) and trimmed using Sickle [98], and the remaining reads were assembled de novo using IDBA-UD v1.1.1 with the “–mink 50, –maxk 92, –steps 8” parameters [99]. To analyze the taxonomic composition of the microorganisms in the four samples, 16S rRNA genes and ribosomal protein S3 (rpS3) were chosen as the marker genes. 16S rRNA genes were identified from the scaffolds using BLASTn [100] (*e* value cutoff is 1e−10) against the SILVA SSU Database v132 [101] and then clustered into OTUs at a similarity cutoff of 95% using USEARCH v10.0.240 [102] (sequences were sorted by length using “sortbylength”command with default parameters and sequences shorter than 700 bp were discarded, then remaining sequences were clustered using cluster_fast command with -id 0.95). A phylogenetic tree of 16S rRNA genes was constructed using the representative 16S rRNA gene sequences and their most related reference sequences (detail parameters can be found in the “Genome binning, taxonomic identification, and phylogeny” section). The rpS3 sequences were identified by BLASTp [100] (*e* value cutoff is 1e−10) against rpS3 sequences retrieved from UniProtKB (https://www.uniprot.org/uniprot/), then the taxonomy was assigned by BLASTp (*e* value cutoff is 1e−10) against the NR database (retrieved on December 2017).

### Genome binning, taxonomic identification, and phylogeny

Binning of the assemblies was performed using MetaBAT v0.32.4 with twelve sets of parameters [103], and the results were then analyzed with Das Tool v1.0 [104] (with search engine of blast and score threshold of 0.1) to obtain optimized metagenomic assembled genomes (MAGs) (command parameters of each set and the evaluation of the results are shown in Figure S12). Subsequently, all the MAGs were further decontaminated using RefineM v0.0.20 with the default settings [42]. The completeness and contamination of the MAGs were calculated using CheckM v1.0.7 [105] with default parameters, and only the MAGs with estimated quality ≥ 50 (defined as completeness—5 × contamination [42]) were considered in the following analyses. To analyze the taxonomy of the MAGs, multiple results were considered, including the taxonomic information from RefineM with the Genome Taxonomy Database (GTDB) [106], taxonomic assignments of the 16S rRNA gene sequences in the MAGs, the phylogeny of concatenated ribosomal proteins, and the phylogeny of the 120 bacterial and 122 archaeal marker genes (principle of the majority). For 16S rRNA genes, the sequences were aligned with those from their corresponding reference genomes using SINA v1.2.11 [107] with default parameters, and columns with more than 5% gaps were trimmed using trimAl v1.4 [108, 109]. For ribosomal protein phylogeny, the sequences of sixteen ribosomal proteins (ribosomal proteins L2, L3, L4, L5, L6, L14, L15, L16, L18, L22, L24, S3, S8, S10, S17, and S19) were extracted from the MAGs [109] and aligned with those from their corresponding reference genomes using MUSCLE v3.8.31 [110] with default parameters, and columns with more than 5% gaps were trimmed using trimAl v1.4 [108, 109]. The reference genomes for ribosomal protein phylogeny were selected from the genomes used by Hug et al. [109]. For the 120 bacterial and 122 archaeal marker genes, those genes of each MAG were extracted and aligned by GTDB-tk v0.3.1 [106] following the official pipeline. The aligned sequences were then concatenated for phylogenetic analysis, and MAGs with less than six ribosomal proteins or ten marker genes were discarded. Phylogenetic trees of the major bacterial taxa (including Bacteroidetes, Nitrospirae, Alpha-, Delta-, and Gammaproteobacteria) were individually constructed with 120 bacterial marker genes of the MAGs for accurate analyses. To identify the closely related MAGs, the pairwise average nucleotide identity (ANI) of MAGs was calculated using the get_homologues package [111] with the default parameters and those with > 70% ANI values were plotted on the phylogenetic trees.

In our study, all of the phylogenetic analyses were performed using RAxML v8.0 [112] on the CIPRES Science Gateway [113]. 1000 bootstraps were performed, and the evolutionary models used were GTRCAT (for nucleotide) and LG+GAMMA (for amino acid). All of the trees were visualized on the iTOL web server [114].

For taxonomic identification of the MAG SZUA-77, the percentages of conserved proteins among Nitrospirae genomes were calculated with the script from https://github.com/hoelzer/pocp [79].

### Gene prediction, annotation, and abundance calculations

Genes of the metagenomic assemblies and MAGs were identified using Prodigal v2.6.3 with the “-p meta” and “-p single” parameters, respectively [115]. Then, they were annotated by combining the results of the eggNOG-mapper v2.0 (with eggNOG v5.0 database) [116] and BLASTp searches (*e* value cutoff is 1e−5) against the NR database (retrieved on December 2017). In particular, to distinguish between *rdsrA* and *dsrA* genes, a phylogenetic tree of these amino acid sequences was built with the reference sequences from Loy et al. [117] and presence of the gene *dsrD* in the same MAG was marked on the tree [118] (Figure S13). The phylogenetic tree of gene *nifH* was built with the reference sequences from Mehta et al. [67] and Cao et al. [72]. Both trees were built with the methods described in the previous section (“Genome binning, taxonomic identification, and phylogeny”). The genes encoding hydrogenases were classified into different functional groups with the reference sequences from Greening et al. [119]. The genes involved in carbohydrate degradation were annotated using the dbcan packages following the pipeline [120], and the subcellular location of the encoded proteins was predicted using PSORTb v3.0.6 following its official pipeline [121].

To calculate the abundance of each scaffold and gene assignment, raw sequencing reads were mapped to the nucleotide sequences of assembled scaffolds using BWA v0.7.5 [122] with the default parameters. The numbers of reads that mapped on the scaffolds and the gene sequences were obtained from the BAM file, and the relative abundances of sequences were calculated with the reads per kilobase per million mapped reads (RPKM) method, which is number of mapped reads/(sequence length × metagenomic size) [43]. The abundances of the metabolisms were calculated with the summation of the genes listed in Table S3. The abundance of 16S rRNA gene and ribosomal protein S3 was calculated with the relative abundance of each sequence. To further examine the coverage of the data, rarefaction analysis was performed with an OTU table of 16S rRNA gene coverage values by using “multiple_rarefactions.py” script in QIIME package [123]. The maximum number of sequences was set to 10,000, and the iterations were set to 10 for each step. The metric multidimensional scaling (MDS) analysis was performed and visualized by plotting the Bray-Curtis distance among the results of taxonomic abundance in our study and reference active and inactive hydrothermal data (Table S5) using vegan package [124].

## Supplementary Information

**Additional file 1:.** Table S1 Information regarding the samples, metagenomes, and assemblies in the current study. Table S2 219 high-quality MAGs (a) and 615 MAGs (b) obtained in the current study. Table S3 Information regarding the functional gene abundance in the samples TVG10 (a), TVG11 (b), TVG12 (c), and TVG13 (d). The abundance of each gene harbored by each taxon is normalized by RPKM (mapped reads per kilobase scaffolds per million sequenced reads) method. Table S4 Information regarding the abundances of CAZymes and secreted CAZymes in the samples TVG10 (a), TVG11 (b), TVG12 (c), and TVG13 (d). The abundance of each gene harbored by each taxon is normalized by RPKM (mapped reads per kilobase scaffolds per million sequenced reads) method. Table S5 Taxonomic details of this study and reference active and inactive samples that were used in the Figure 2 and S7b. Table S6 The accession ID and taxonomic information of the NifH sequences used in Figure 5a. Supplementary Data 1 Phylogenetic tree of bacterial MAGs based on 120 bacterial marker genes in newick format. The values depicted on the branches are bootstrap values. Supplementary Data 2 Phylogenetic tree of archaeal MAGs based on 122 archaeal marker genes in newick format. The values depicted on the branches are bootstrap values. Supplementary Data 3 Phylogenetic tree of 16S rRNA genes in newick format. The values depicted on the branches are bootstrap values. Supplementary Data 4 Phylogenetic tree of MAGs based on 16 ribosomal protein sequences in newick format. The values depicted on the branches are bootstrap values.

**Additional file 2: **Fig. S1 Bathymetric map of the southern Mid-Atlantic Ocean and the location of the Deyin-1 hydrothermal field. Fig. S2 Phylogenetic tree and relative abundance of 16S rRNA genes in the current study. The maximum likelihood phylogenetic tree was constructed based on 16S rRNA gene sequences from the assembled scaffolds. The scale bar represents 0.1 nucleotide substitutions per sequence position. The bar plots are based on the relative abundance of each sequence, and the color of the bars represents the samples that each sequence was obtained from. The full phylogenetic tree is available in Supplementary Data 3. Fig. S3 Ribosomal protein tree of the MAGs in the current study. The maximum likelihood phylogenetic tree was constructed based on 16 ribosomal protein sequences. The scale bar represents 0.1 amino acid substitutions per sequence position. The colors represent the taxonomy of the MAG. The full phylogenetic tree is available in Supplementary Data 4. Fig. S4 Phylogenetic tree, relative abundance, and functional potentials of Alphaproteobacteria MAGs. The maximum likelihood phylogenetic tree was constructed based on 16 concatenated ribosomal protein sequences. The scale bar represents 0.1 amino acid substitutions per sequence position. RPKM value is the relative abundance of each MAG, calculated by number of mapped reads / (sequence length × metagenomic size). Fig. S5 Phylogenetic tree, relative abundance, and functional potentials of Gammaproteobacteria MAGs. The maximum likelihood phylogenetic tree was constructed based on 16 concatenated ribosomal protein sequences. The scale bar represents 0.1 amino acid substitutions per sequence position. RPKM value is the relative abundance of each MAG, calculated by number of mapped reads / (sequence length × metagenomic size). Fig. S6 Phylogenetic tree, relative abundance, and functional potentials of Bacteroidetes MAGs. The maximum likelihood phylogenetic tree was constructed based on 16 concatenated ribosomal protein sequences. The scale bar represents 0.1 amino acid substitutions per sequence position. RPKM value is the relative abundance of each MAG, calculated by number of mapped reads / (sequence length × metagenomic size). Fig. S7 Venn diagram of OTUs clustered with 16S rRNA gene sequences in this study (a) and MDS analysis of the taxonomic compositions with reference active and inactive chimney data (b). The taxonomic details of reference samples were available in Table S5. Fig. S8 Rarefaction curves based on the 16S rRNA gene coverages in all samples. (a) Observed OTU number. (b) Chao1 index. Fig. S9 Relative abundance of functional genes encoded by the taxa in each sample. The details of gene abundance are available in Table S3. Fig. S10 Relative abundance of carbohydrate-active enzymes (CAZymes) encoded by the taxa in each sample. The purple triangle indicates CAZymes with a potential secretion signal. The details of CAZymes abundance are available in Table S4. Fig. S11 Phylogenetic tree based on 120 bacterial marker genes (a) and the percentages of conserved proteins (b) with the reference Nitrospirae genomes and the MAG SZUA-77. Fig. S12 Command parameters of twelve binning methods using MetaBAT (a) and the evaluation of each result comparing to the final result optimized by using DAS_Tool. Fig. S13 Phylogenetic tree of *dsrA* and *rdsrA* amino acid sequences from the MAGs. The scale bar of the phylogenetic trees represents 0.1 amino acid substitutions per sequence position. The bar plots are based on the average abundance of the sequences. The stars represent the existence of *dsrD* gene in the same MAG.

## Data Availability

All sequence data and sample information are available at NCBI under BioProject ID PRJNA385762. The accession numbers for the MAGs are provided in Table S2.

## Abbreviations

16S: 16S rRNA gene
ANI: Average nucleotide identity
*aprAB*: Adenylylsulfate reductase
CAZymes: Carbohydrate-active enzymes
CBB: Calvin-Benson-Bassham
*ccoNOP*: cbb3-type terminal cytochrome c oxidase
*coxABC*: Cytochrome c oxidase
CPR: Candidate Phyla Radiation
*cydAB*: Cytochrome d oxidase
*cyoABCDE*: Cytochrome o oxidase
*dsr*: Dissimilatory sulfite reductase
GTDB: Genome Taxonomy Database
ITSNTS: It’s the song not the singer
MAG: Metagenomic assembled genomes
MOR: Mid-ocean ridges
*nifH*: Nitrogenase iron protein
OTU: Operational taxonomic unit
*rdsrAB*: Reverse dissimilatory sulfite reductase
rps3: Ribosomal protein S3
RPKM: Reads per kilobase per million mapped reads
rTCA: Reverse tricarboxylic acid
*sat*: Sulfate adenylyltransferase
SOX: Sulfur oxidation complex
