# Correction to: Genome-resolved evidence for functionally redundant communities and novel nitrogen fixers in the deyin-1 hydrothermal field, Mid-Atlantic Ridge

**DOI:** 10.1186/s40168-022-01253-8

**Published:** 2022-03-05

**Authors:** Jie Pan, Wei Xu, Zhichao Zhou, Zongze Shao, Chunming Dong, Lirui Liu, Zhuhua Luo, Meng Li

**Affiliations:** 1 Archaeal Biology Center, Shenzhen Key Laboratory of Marine Microbiome Engineering, Institute for Advanced Study, Shenzhen University, Shenzhen, Guangdong P. R. China; 2 Key Laboratory of Marine Biogenetic Resources, Third Institute of Oceanography, Ministry of Natural Resources, Xiamen, Fujian P. R. China; 3 Department of Bacteriology, University of Wisconsin-Madison, Madison, WI 53706 USA; 4 School of Marine Sciences, Nanjing University of Information Science & Technology, Nanjing, 210044 P. R. China

**Correction to: Microbiome 10, 8 (2022)**

**https://doi.org/10.1186/s40168-021-01202-x**

Following publication of the original article [1], the authors reported an error in Contributions section. Samples were collected by the authors ZS and CD, WX performed the laboratory works.

From: "JP, ML, and ZL designed the experiment; WX collected the samples, with the contribution of ZS and CD; JP and ZC performed the informatics work; JP, LL, and ML wrote the paper, with the contributions from all authors."

To: "JP, ML, and ZL designed the experiment; ZS and CD collected the samples; WX performed the laboratory works; JP and ZC performed the informatics works; JP, LL, and ML wrote the paper, with the contributions from all authors."

The authors apologize for this error.
