# Supplementary material for: Genome-resolved evidence for functionally redundant communities and novel nitrogen fixers in the deyin-1 hydrothermal field, Mid-Atlantic Ridge
Source: Microbiome. 2022 Jan 19;10:8. doi: 10.1186/s40168-021-01202-x (PMC8767757; doi:10.1186/s40168-021-01202-x)
Supplement: Supplementary file 3 — Additional file 2: Fig. S1 Bathymetric map of the southern Mid-Atlantic Ocean and the location of the Deyin-1 hydrothermal field. Fig. S2 Phylogenetic tree and relative abundance of 16S rRNA genes in the current study. The maximum likelihood phylogenetic tree was constructed based on 16S rRNA gene sequences from the assembled scaffolds. The scale bar represents 0.1 nucleotide substitutions per sequence position. The bar plots are based on the relative abundance of each sequence, and the color of the bars represents the samples that each sequence was obtained from. The full phylogenetic tree is available in Supplementary Data 3. Fig. S3 Ribosomal protein tree of the MAGs in the current study. The maximum likelihood phylogenetic tree was constructed based on 16 ribosomal protein sequences. The scale bar represents 0.1 amino acid substitutions per sequence position. The colors represent the taxonomy of the MAG. The full phylogenetic tree is available in Supplementary Data 4. Fig. S4 Phylogenetic tree, relative abundance, and functional potentials of Alphaproteobacteria MAGs. The maximum likelihood phylogenetic tree was constructed based on 16 concatenated ribosomal protein sequences. The scale bar represents 0.1 amino acid substitutions per sequence position. RPKM value is the relative abundance of each MAG, calculated by number of mapped reads / (sequence length × metagenomic size). Fig. S5 Phylogenetic tree, relative abundance, and functional potentials of Gammaproteobacteria MAGs. The maximum likelihood phylogenetic tree was constructed based on 16 concatenated ribosomal protein sequences. The scale bar represents 0.1 amino acid substitutions per sequence position. RPKM value is the relative abundance of each MAG, calculated by number of mapped reads / (sequence length × metagenomic size). Fig. S6 Phylogenetic tree, relative abundance, and functional potentials of Bacteroidetes MAGs. The maximum likelihood phylogenetic tree was constructed based on 16 concatenated r [file 40168_2021_1202_MOESM3_ESM.pdf]

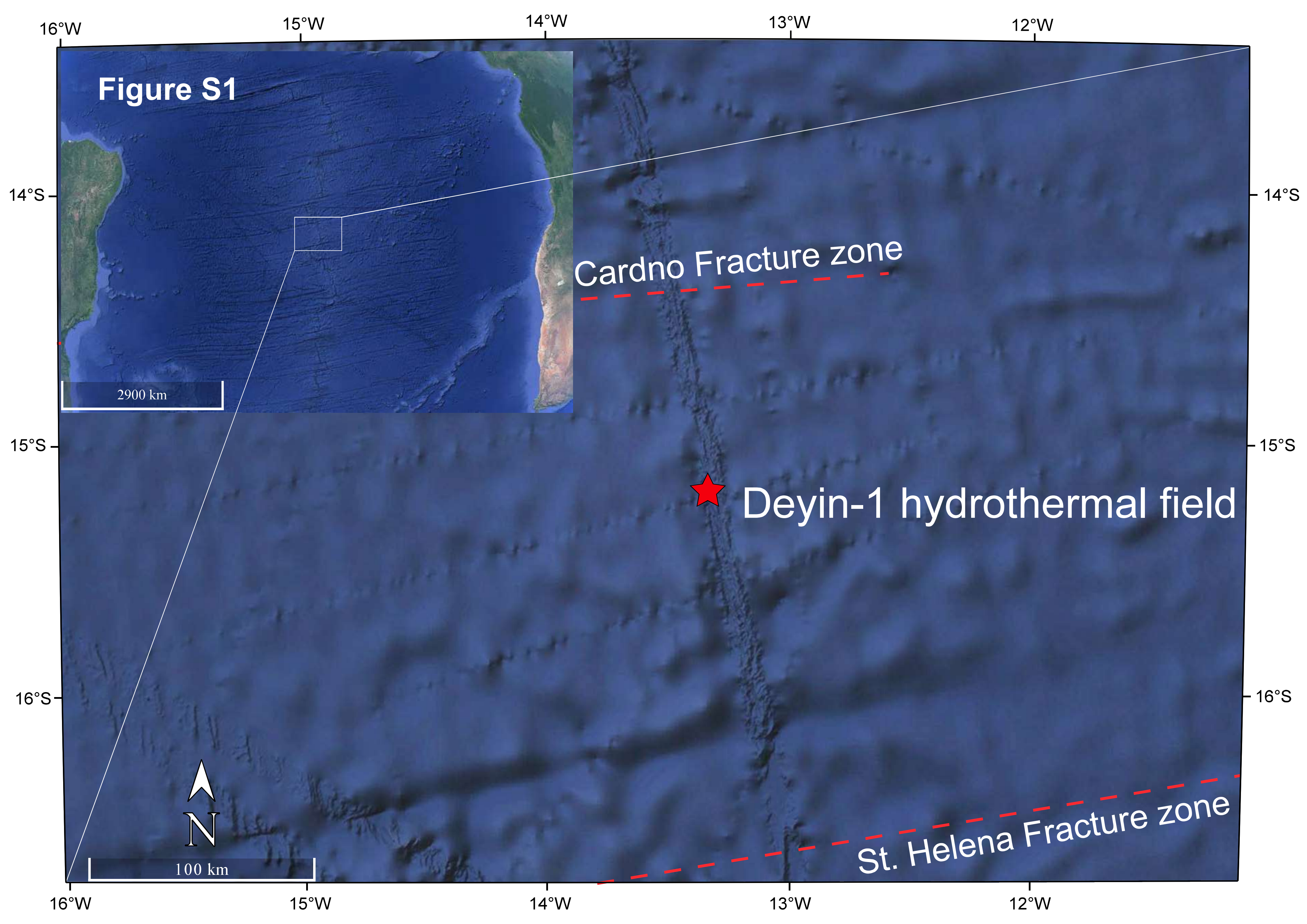
Figure S1
16°W
15°W
14°W
13°W
12°W
14°S
15°S
16°S
2900 km
Cardno Fracture zone
Deyin-1 hydrothermal field
St. Helena Fracture zone
N
100 km

Figure S2

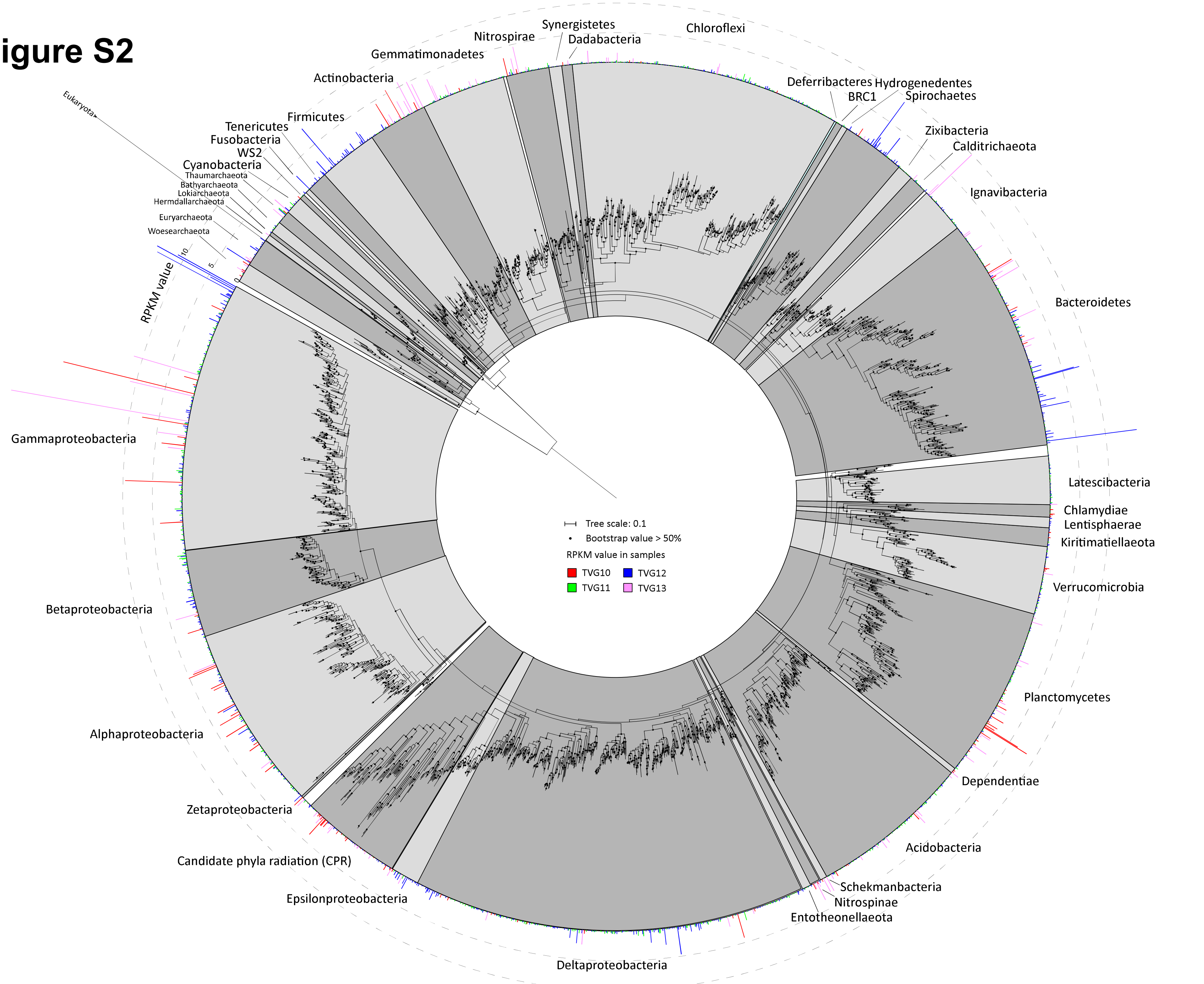

Eukaryota
RPKM value
0
5
10
Woesearchaeota
Euryarchaeota
Hermdallarchaeota
Lokiarchaeota
Bathyarchaeota
Thaumarchaeota
Cyanobacteria
WS2
Fusobacteria
Tenericutes
Firmicutes
Actinobacteria
Gemmatimonadetes
Nitrospirae
Synergistetes
Dadabacteria
Chloroflexi
Deferribacteres
BRC1
Hydrogenedentes
Spirochaetes
Zixibacteria
Calditrichaeota
Ignavibacteria
Bacteroidetes
Latescibacteria
Chlamydiae
Lentisphaerae
Kiritimatiellaeota
Verrucomicrobia
Planctomycetes
Dependentiae
Acidobacteria
Schekmanbacteria
Nitrospinae
Entotheonellaeota
Deltaproteobacteria
Epsilonproteobacteria
Candidate phyla radiation (CPR)
Zetaproteobacteria
Alphaproteobacteria
Betaproteobacteria
Gammaproteobacteria
Tree scale: 0.1
Bootstrap value > 50%
RPKM value in samples
TVG10
TVG11
TVG12
TVG13

Figure S3

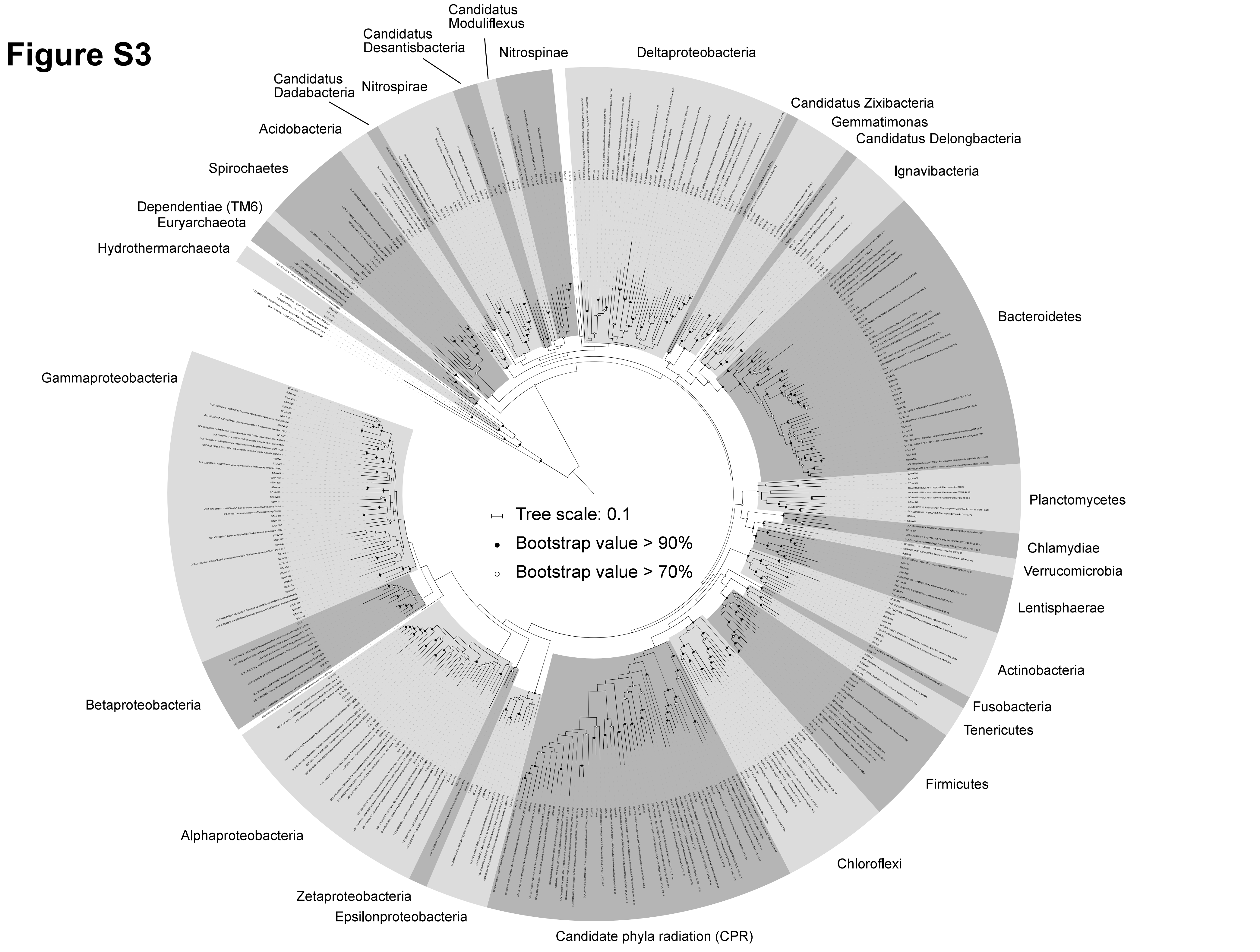

Candidatus Moduliflexus
Candidatus Desantisbacteria
Nitrospinae
Deltaproteobacteria
Candidatus Dadabacteria
Nitrospirae
Candidatus Zixibacteria
Gemmatimonas
Candidatus Delongbacteria
Acidobacteria
Spirochaetes
Ignavibacteria
Dependentiae (TM6)
Euryarchaeota
Hydrothermarchaeota
Bacteroidetes
Gammaproteobacteria
Planctomycetes
Tree scale: 0.1
Bootstrap value > 90%
Bootstrap value > 70%
Chlamydiae
Verrucomicrobia
Lentisphaerae
Actinobacteria
Betaproteobacteria
Fusobacteria
Tenericutes
Firmicutes
Alphaproteobacteria
Chloroflexi
Zetaproteobacteria
Epsilonproteobacteria
Candidate phyla radiation (CPR)

Figure S4

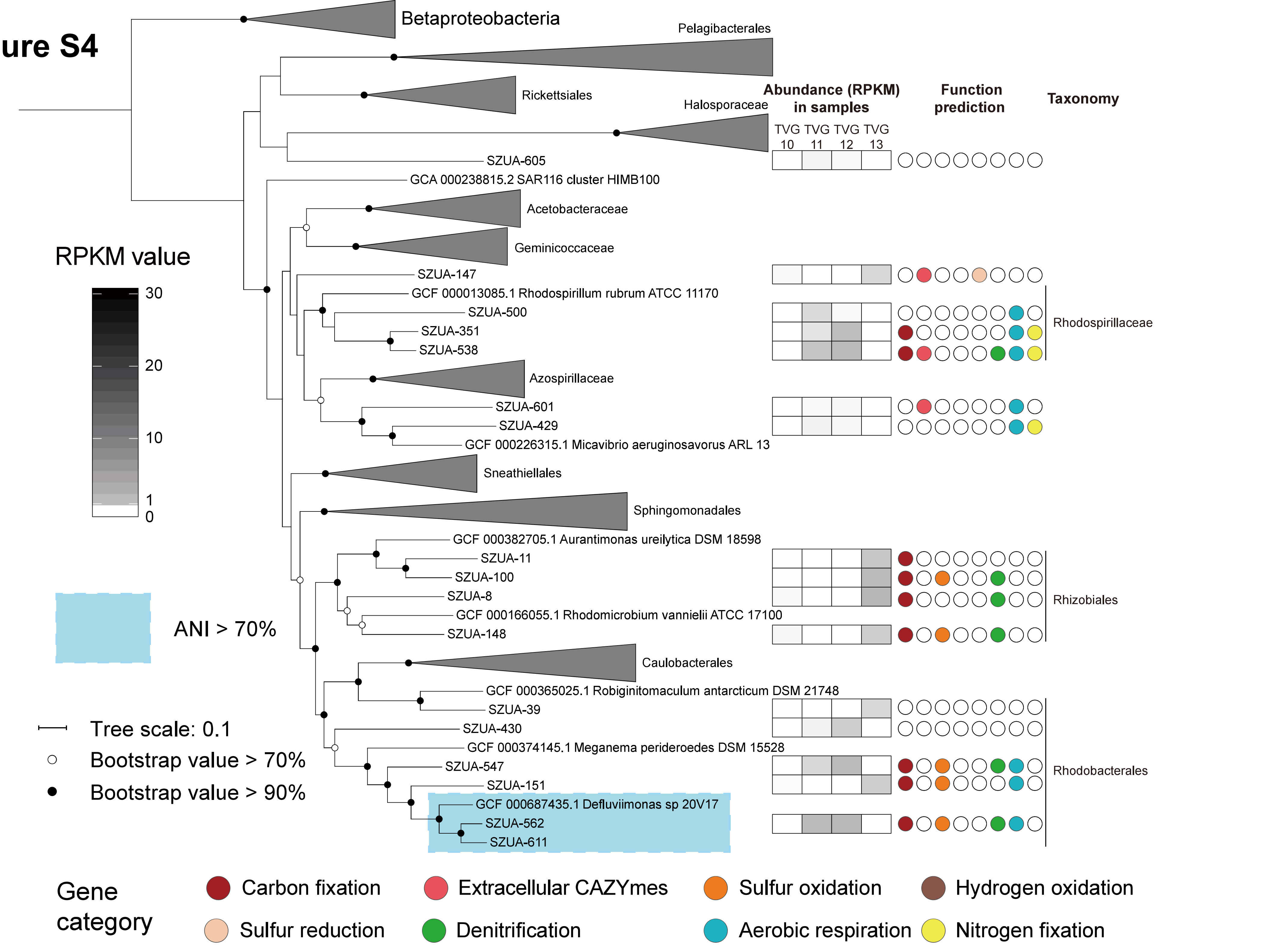
Betaproteobacteria
Pelagibacterales
Rickettsiales
Halosporaceae
Abundance (RPKM) in samples
TVG 10
TVG 11
TVG 12
TVG 13
Function prediction
Taxonomy
SZUA-605
GCA 000238815.2 SAR116 cluster HIMB100
Acetobacteraceae
Geminicoccaceae
SZUA-147
GCF 000013085.1 Rhodospirillum rubrum ATCC 11170
SZUA-500
SZUA-351
SZUA-538
Rhodospirillaceae
Azospirillaceae
SZUA-601
SZUA-429
GCF 000226315.1 Micavibrio aeruginosavorus ARL 13
Sneathiellales
Sphingomonadales
GCF 000382705.1 Aurantimonas ureilytica DSM 18598
SZUA-11
SZUA-100
SZUA-8
GCF 000166055.1 Rhodomicrobium vannielii ATCC 17100
SZUA-148
Rhizobiales
Caulobacterales
GCF 000365025.1 Robiginitomaculum antarcticum DSM 21748
SZUA-39
SZUA-430
GCF 000374145.1 Meganema perideroedes DSM 15528
SZUA-547
SZUA-151
GCF 000687435.1 Defluviimonas sp 20V17
SZUA-562
SZUA-611
Rhodobacterales
RPKM value
30
20
10
1
0
ANI > 70%
Tree scale: 0.1
Bootstrap value > 70%
Bootstrap value > 90%
Gene category
Carbon fixation
Extracellular CAZYmes
Sulfur oxidation
Hydrogen oxidation
Sulfur reduction
Denitrification
Aerobic respiration
Nitrogen fixation

Figure S5

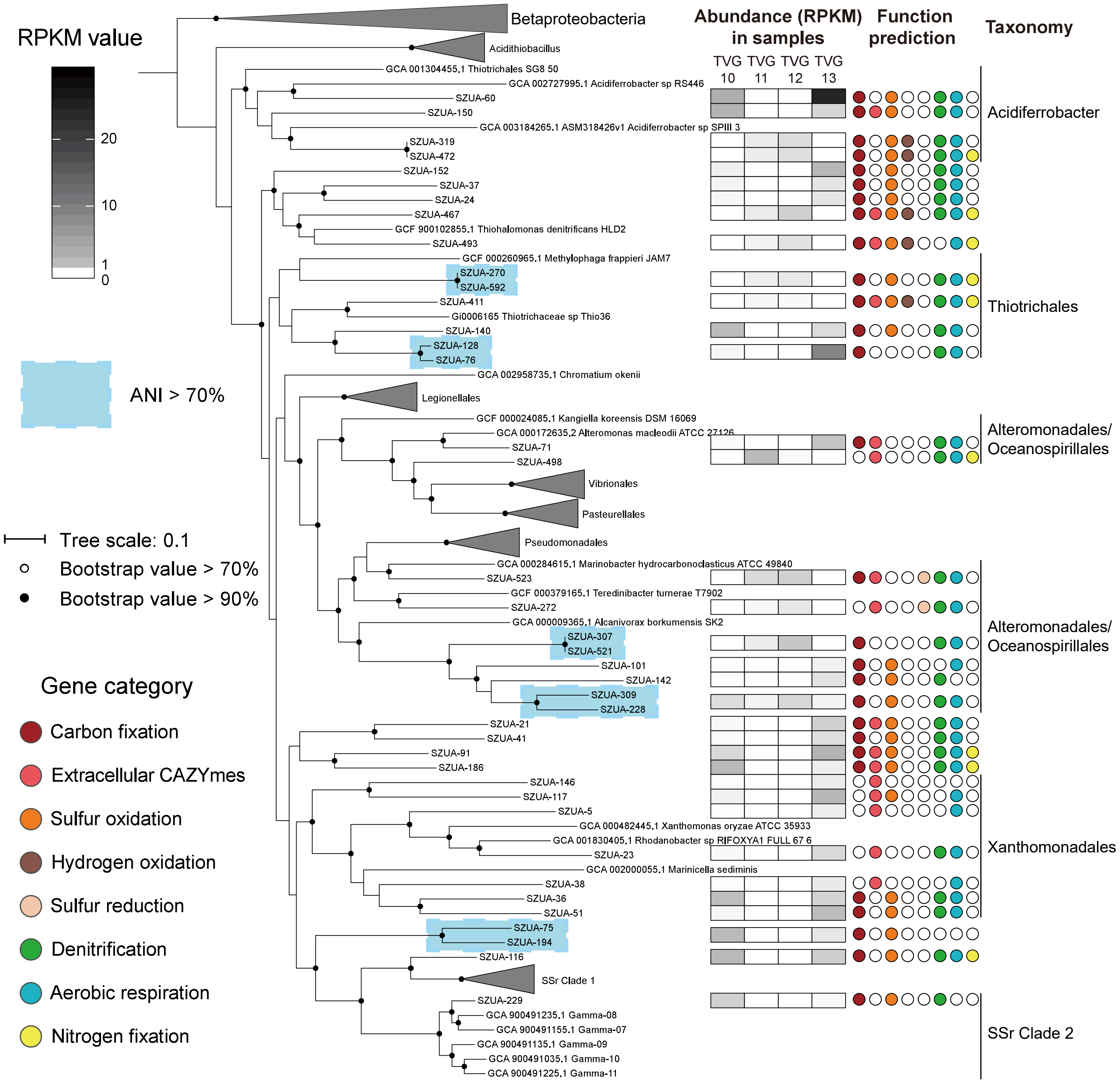

RPKM value
20
10
1
0
ANI > 70%
Tree scale: 0.1
Bootstrap value > 70%
Bootstrap value > 90%
Gene category
Carbon fixation
Extracellular CAZYmes
Sulfur oxidation
Hydrogen oxidation
Sulfur reduction
Denitrification
Aerobic respiration
Nitrogen fixation
Betaproteobacteria
Acidithiobacillus
GCA 001304455.1 Thiotrichales SG8 50
GCA 002727995.1 Acidiferrobacter sp RS446
SZUA-60
SZUA-150
GCA 003184265.1 ASM318426v1 Acidiferrobacter sp SPIII 3
SZUA-319
SZUA-472
SZUA-152
SZUA-37
SZUA-24
SZUA-467
GCF 900102855.1 Thiohalomonas denitrificans HLD2
SZUA-493
GCF 000260965.1 Methylophaga frappieri JAM7
SZUA-270
SZUA-592
SZUA-411
Gi0006165 Thiotrichaceae sp Thio36
SZUA-140
SZUA-128
SZUA-76
GCA 002958735.1 Chromatium okenii
Legionellales
GCF 000024085.1 Kangiella koreensis DSM 16069
GCA 000172635.2 Alteromonas macleodii ATCC 27126
SZUA-71
SZUA-498
Vibrionales
Pasteurellales
Pseudomonadales
GCA 000284615.1 Marinobacter hydrocarbonoclasticus ATCC 49840
SZUA-523
GCF 000379165.1 Teredinibacter turnerae T7902
SZUA-272
GCA 000009365.1 Alcanivorax borkumensis SK2
SZUA-307
SZUA-521
SZUA-101
SZUA-142
SZUA-309
SZUA-228
SZUA-21
SZUA-41
SZUA-91
SZUA-186
SZUA-146
SZUA-117
SZUA-5
GCA 000482445.1 Xanthomonas oryzae ATCC 35933
GCA 001830405.1 Rhodanobacter sp RIFOXYA1 FULL 67 6
SZUA-23
GCA 002000055.1 Marinicella sediminis
SZUA-38
SZUA-36
SZUA-51
SZUA-75
SZUA-194
SZUA-116
SSr Clade 1
SZUA-229
GCA 900491235.1 Gamma-08
GCA 900491155.1 Gamma-07
GCA 900491135.1 Gamma-09
GCA 900491035.1 Gamma-10
GCA 900491225.1 Gamma-11
Abundance (RPKM) in samples
TVG 10
TVG 11
TVG 12
TVG 13
Function prediction
Taxonomy
Acidiferrobacter
Thiotrichales
Alteromonadales/ Oceanospirillales
Alteromonadales/ Oceanospirillales
Xanthomonadales
SSr Clade 2

Figure S6

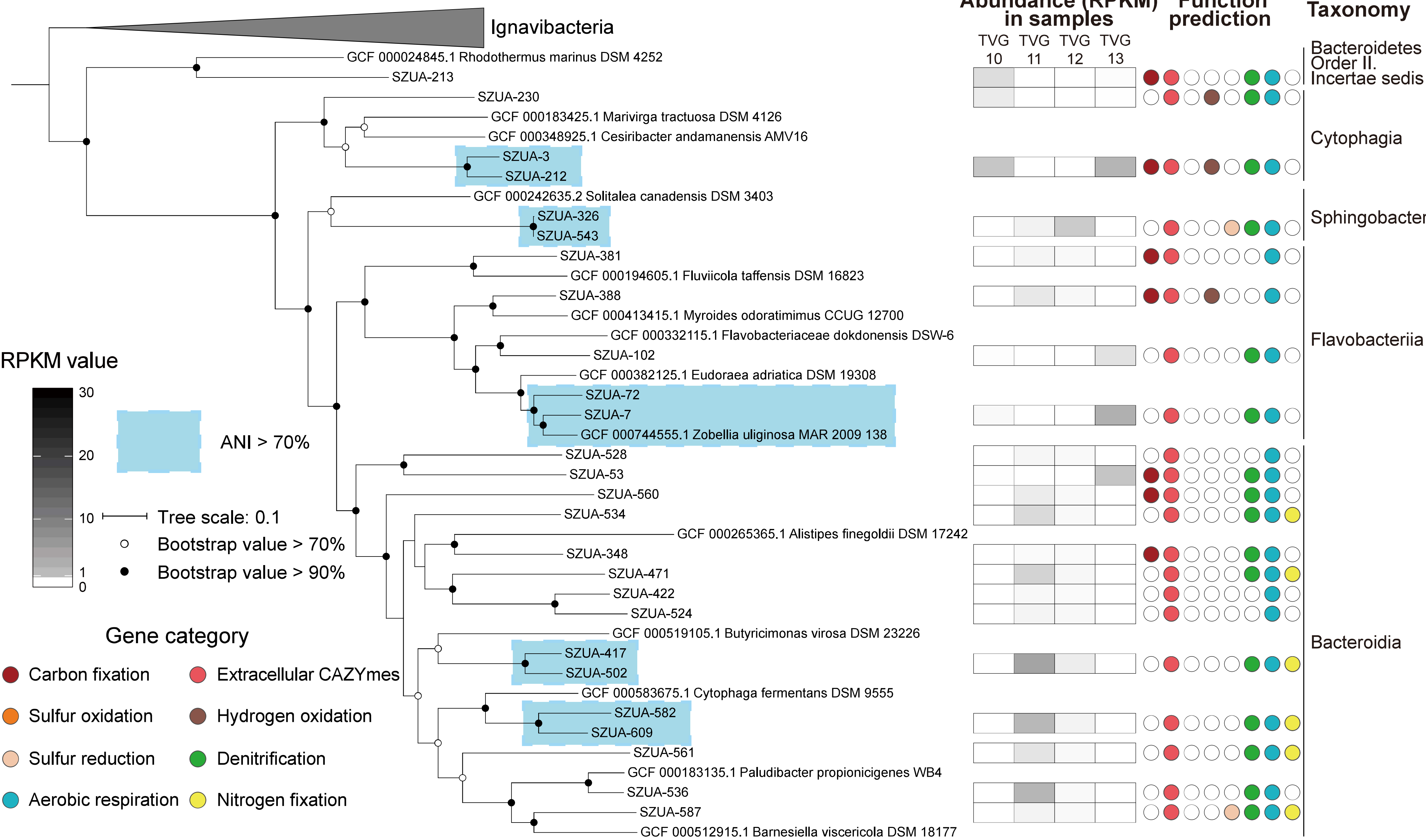
Ignavibacteria
GCF 000024845.1 Rhodothermus marinus DSM 4252
SZUA-213
SZUA-230
GCF 000183425.1 Marivirga tractuosa DSM 4126
GCF 000348925.1 Cesiribacter andamanensis AMV16
SZUA-3
SZUA-212
GCF 000242635.2 Solitalea canadensis DSM 3403
SZUA-326
SZUA-543
SZUA-381
GCF 000194605.1 Fluviicola taffensis DSM 16823
SZUA-388
GCF 000413415.1 Myroides odoratimimus CCUG 12700
GCF 000332115.1 Flavobacteriaceae dokdonensis DSW-6
SZUA-102
GCF 000382125.1 Eudoraea adriatica DSM 19308
SZUA-72
SZUA-7
GCF 000744555.1 Zobellia uliginosa MAR 2009 138
SZUA-528
SZUA-53
SZUA-560
SZUA-534
GCF 000265365.1 Alistipes finegoldii DSM 17242
SZUA-348
SZUA-471
SZUA-422
SZUA-524
GCF 000519105.1 Butyricimonas virosa DSM 23226
SZUA-417
SZUA-502
GCF 000583675.1 Cytophaga fermentans DSM 9555
SZUA-582
SZUA-609
SZUA-561
GCF 000183135.1 Paludibacter propionicigenes WB4
SZUA-536
SZUA-587
GCF 000512915.1 Barnesiella viscericola DSM 18177
Abundance (RPKM) in samples
TVG 10
TVG 11
TVG 12
TVG 13
Function prediction
Taxonomy
Bacteroidetes Order II. Incertae sedis
Cytophagia
Sphingobacter
Flavobacteriia
Bacteroidia
RPKM value
30
20
10
1
0
ANI > 70%
Tree scale: 0.1
Bootstrap value > 70%
Bootstrap value > 90%
Gene category
Carbon fixation
Extracellular CAZYmes
Sulfur oxidation
Hydrogen oxidation
Sulfur reduction
Denitrification
Aerobic respiration
Nitrogen fixation

Figure S7

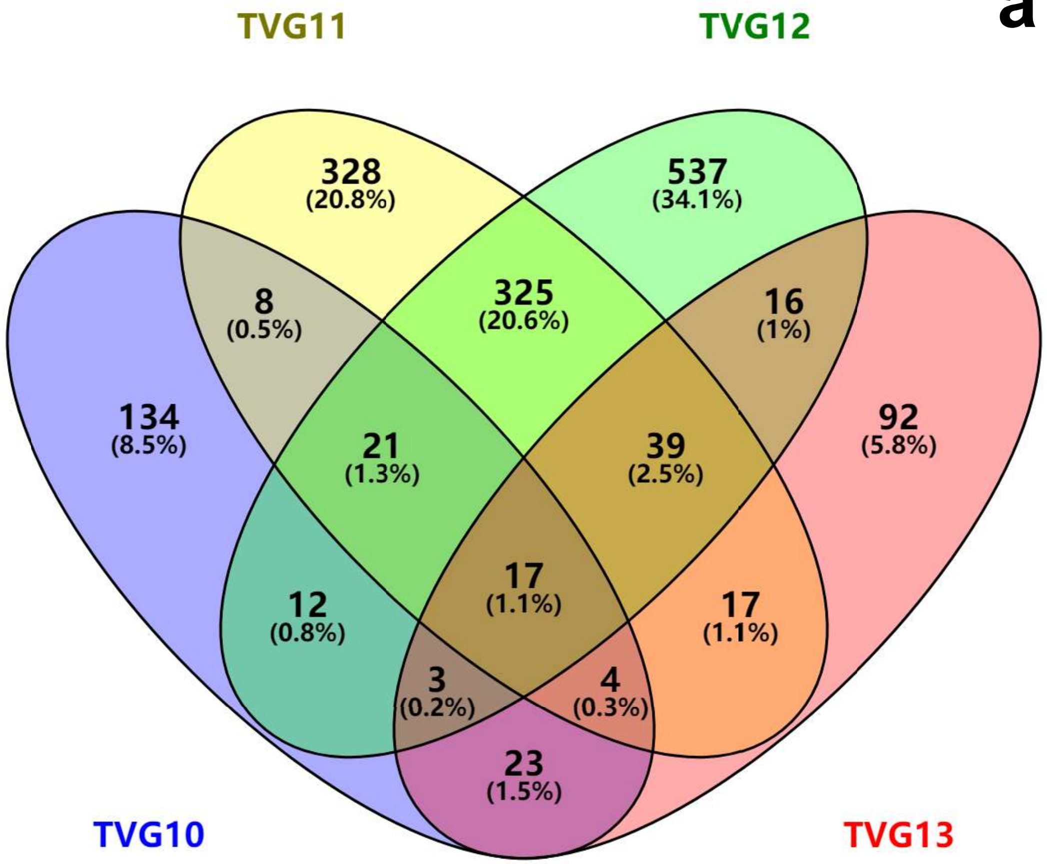
a
TVG11
TVG12
TVG10
TVG13
328
(20.8%)
537
(34.1%)
8
(0.5%)
325
(20.6%)
16
(1%)
134
(8.5%)
21
(1.3%)
39
(2.5%)
92
(5.8%)
17
(1.1%)
12
(0.8%)
17
(1.1%)
3
(0.2%)
4
(0.3%)
23
(1.5%)

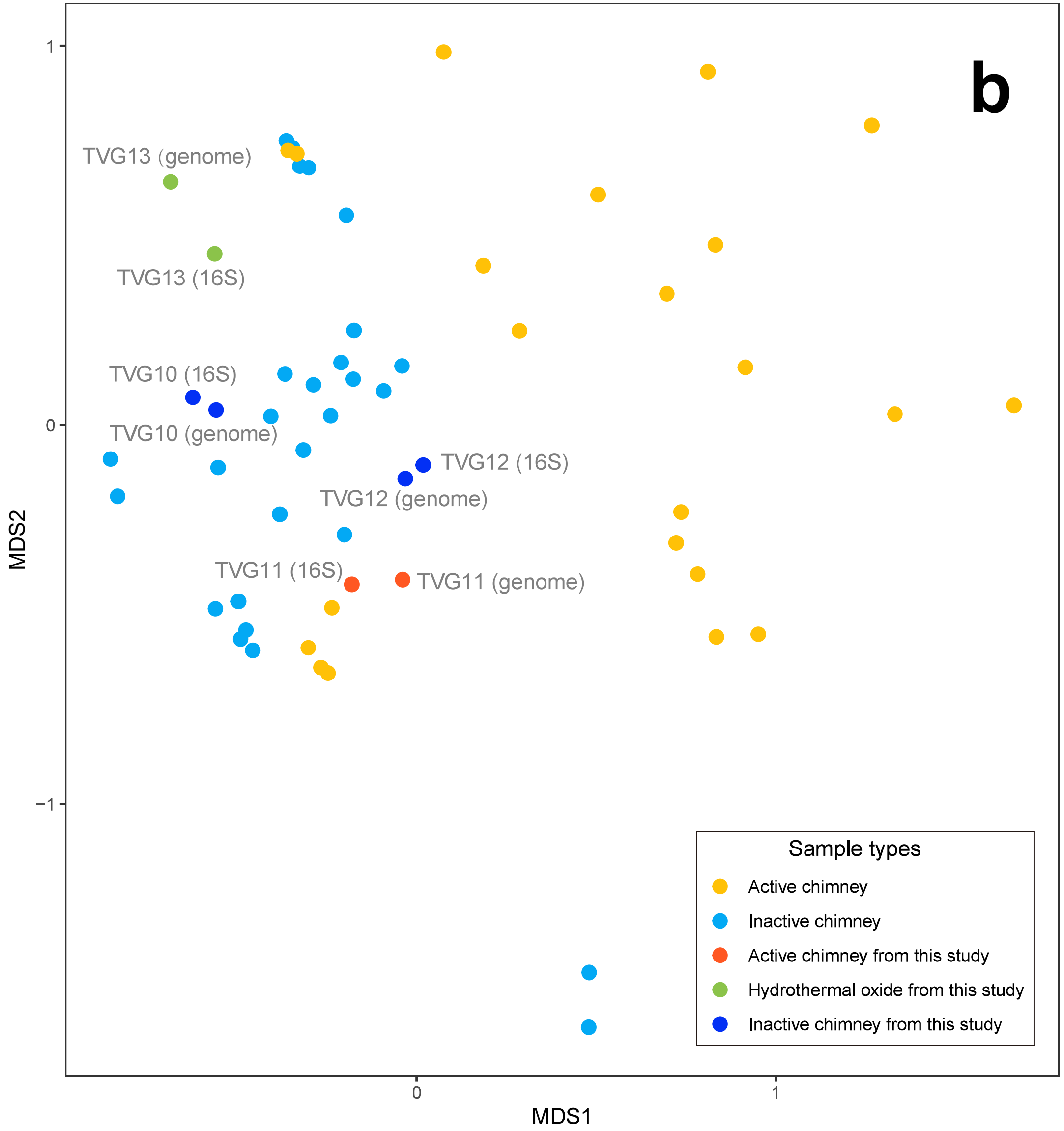
b
TVG13 (genome)
TVG13 (16S)
TVG10 (16S)
TVG10 (genome)
TVG12 (16S)
TVG12 (genome)
TVG11 (16S)
TVG11 (genome)
MDS2
MDS1
1
0
−1
0
1
Sample types
Active chimney
Inactive chimney
Active chimney from this study
Hydrothermal oxide from this study
Inactive chimney from this study

Figure S8

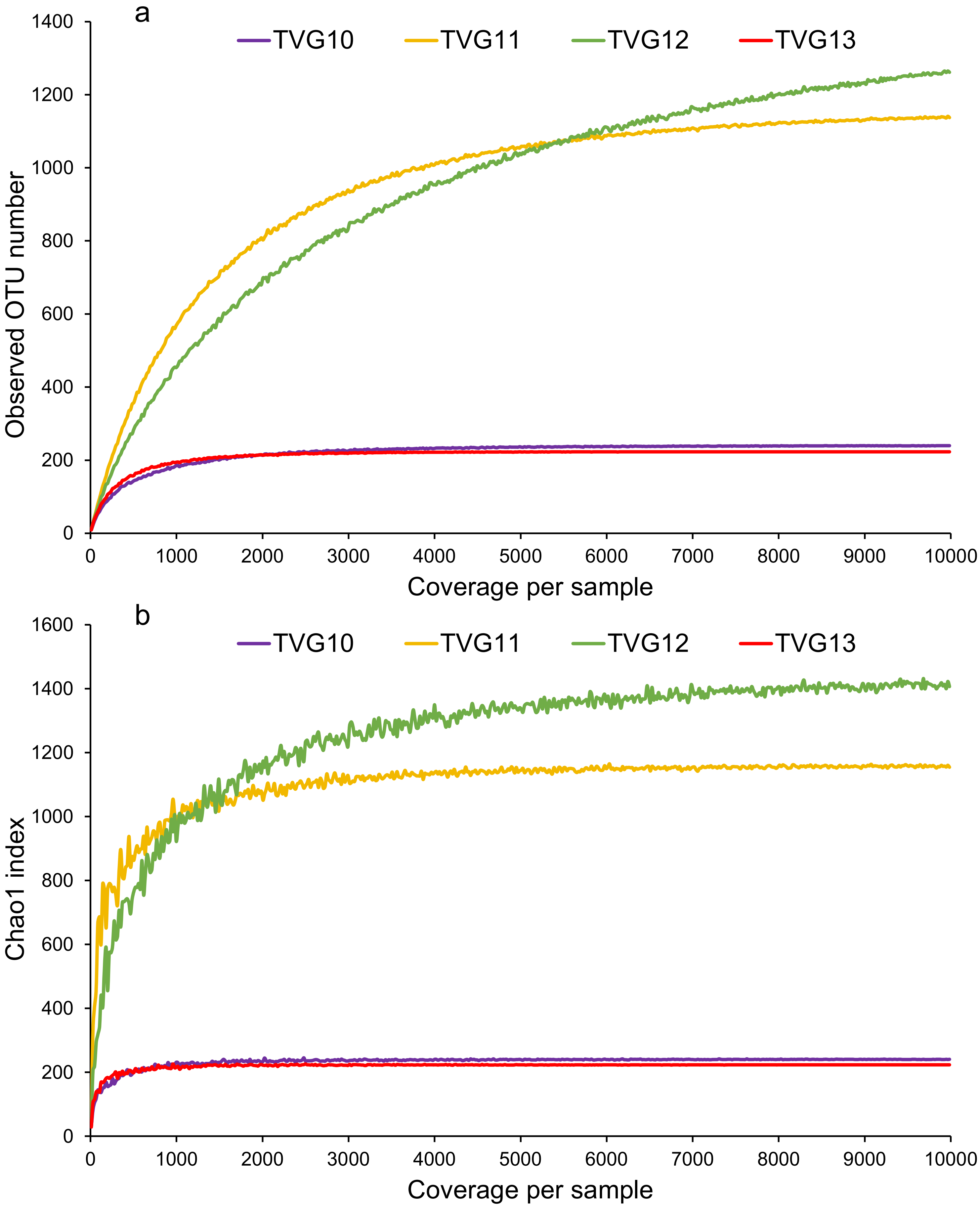
a
TVG10
TVG11
TVG12
TVG13
Observed OTU number
0
200
400
600
800
1000
1200
1400
0
1000
2000
3000
4000
5000
6000
7000
8000
9000
10000
Coverage per sample
b
TVG10
TVG11
TVG12
TVG13
Chao1 index
0
200
400
600
800
1000
1200
1400
1600
0
1000
2000
3000
4000
5000
6000
7000
8000
9000
10000
Coverage per sample

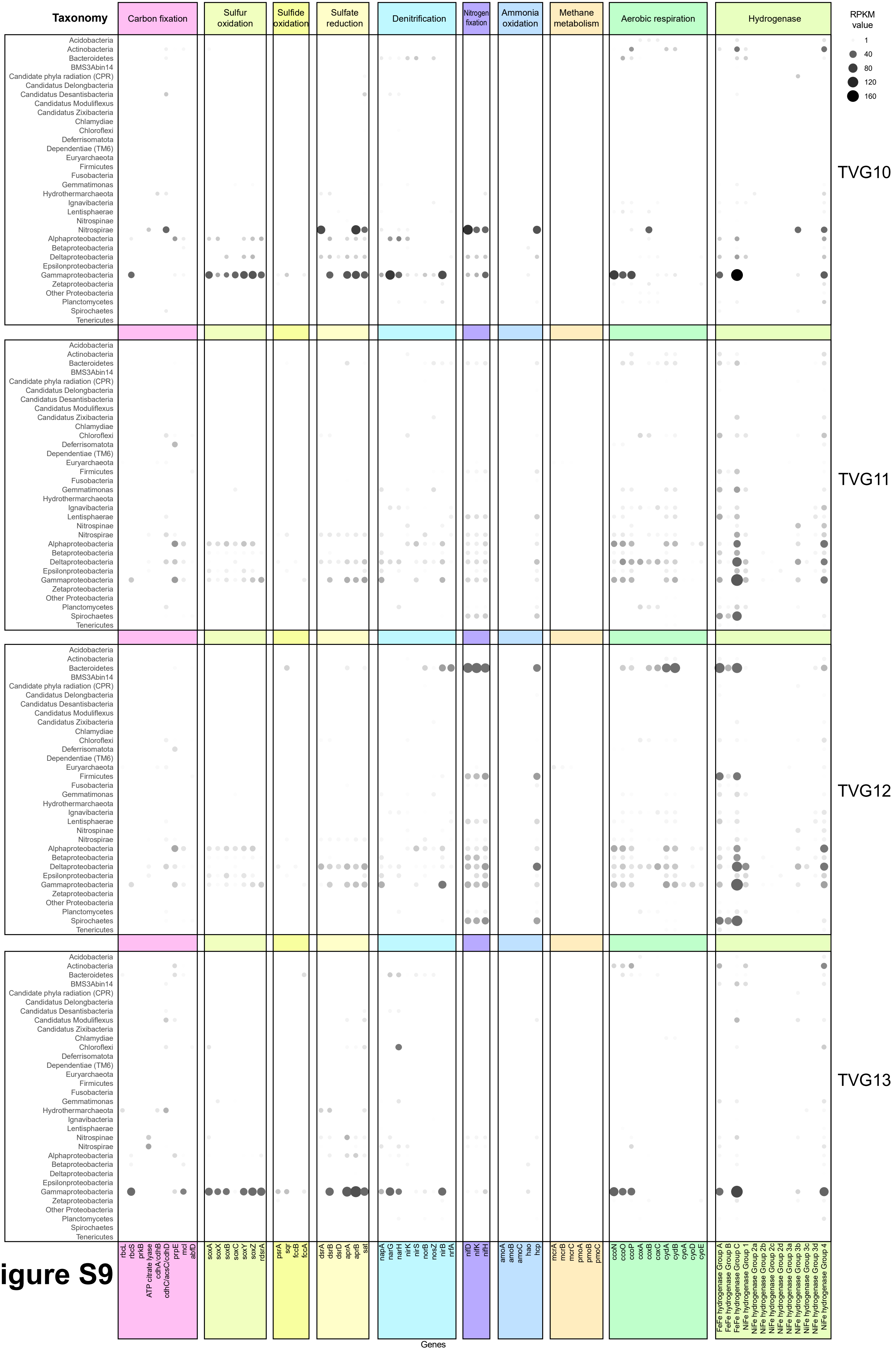
Taxonomy
Carbon fixation
Sulfur oxidation
Sulfide oxidation
Sulfate reduction
Denitrification
Nitrogen fixation
Ammonia oxidation
Methane metabolism
Aerobic respiration
Hydrogenase
RPKM value
1
40
80
120
160
Acidobacteria
Actinobacteria
Bacteroidetes
BMS3Abin14
Candidate phyla radiation (CPR)
Candidatus Delongbacteria
Candidatus Desantisbacteria
Candidatus Moduliflexus
Candidatus Zixibacteria
Chlamydiae
Chloroflexi
Deferrisomatota
Dependentiae (TM6)
Euryarchaeota
Firmicutes
Fusobacteria
Gemmatimonas
Hydrothermarchaeota
Ignavibacteria
Lentisphaerae
Nitrospinae
Nitrospirae
Alphaproteobacteria
Betaproteobacteria
Deltaproteobacteria
Epsilonproteobacteria
Gammaproteobacteria
Zetaproteobacteria
Other Proteobacteria
Planctomycetes
Spirochaetes
Tenericutes
TVG10
TVG11
TVG12
TVG13
rbcL
rbcS
prkB
ATP citrate lyase
cdhA/cdhB
cdhC/acsC/cdhD
prpE
mcl
abfD
soxA
soxX
soxB
soxC
soxY
soxZ
rdsrA
psrA
sqr
fccB
fccA
dsrA
dsrB
dsrD
aprA
aprB
sat
napA
narG
narH
nirK
nirS
norB
nosZ
nirB
nrfA
nifD
nifK
nifH
amoA
amoB
amoC
hao
hcp
mcrA
mcrB
mcrC
pmoA
pmoB
pmoC
ccoN
ccoO
ccoP
coxA
coxB
coxC
cydA
cydB
cyoA
cyoD
cyoE
FeFe hydrogenase Group A
FeFe hydrogenase Group B
FeFe hydrogenase Group C
NiFe hydrogenase Group 1
NiFe hydrogenase Group 2a
NiFe hydrogenase Group 2b
NiFe hydrogenase Group 2c
NiFe hydrogenase Group 2d
NiFe hydrogenase Group 3a
NiFe hydrogenase Group 3b
NiFe hydrogenase Group 3c
NiFe hydrogenase Group 3d
NiFe hydrogenase Group 4
Genes
Figure S9

Figure S10

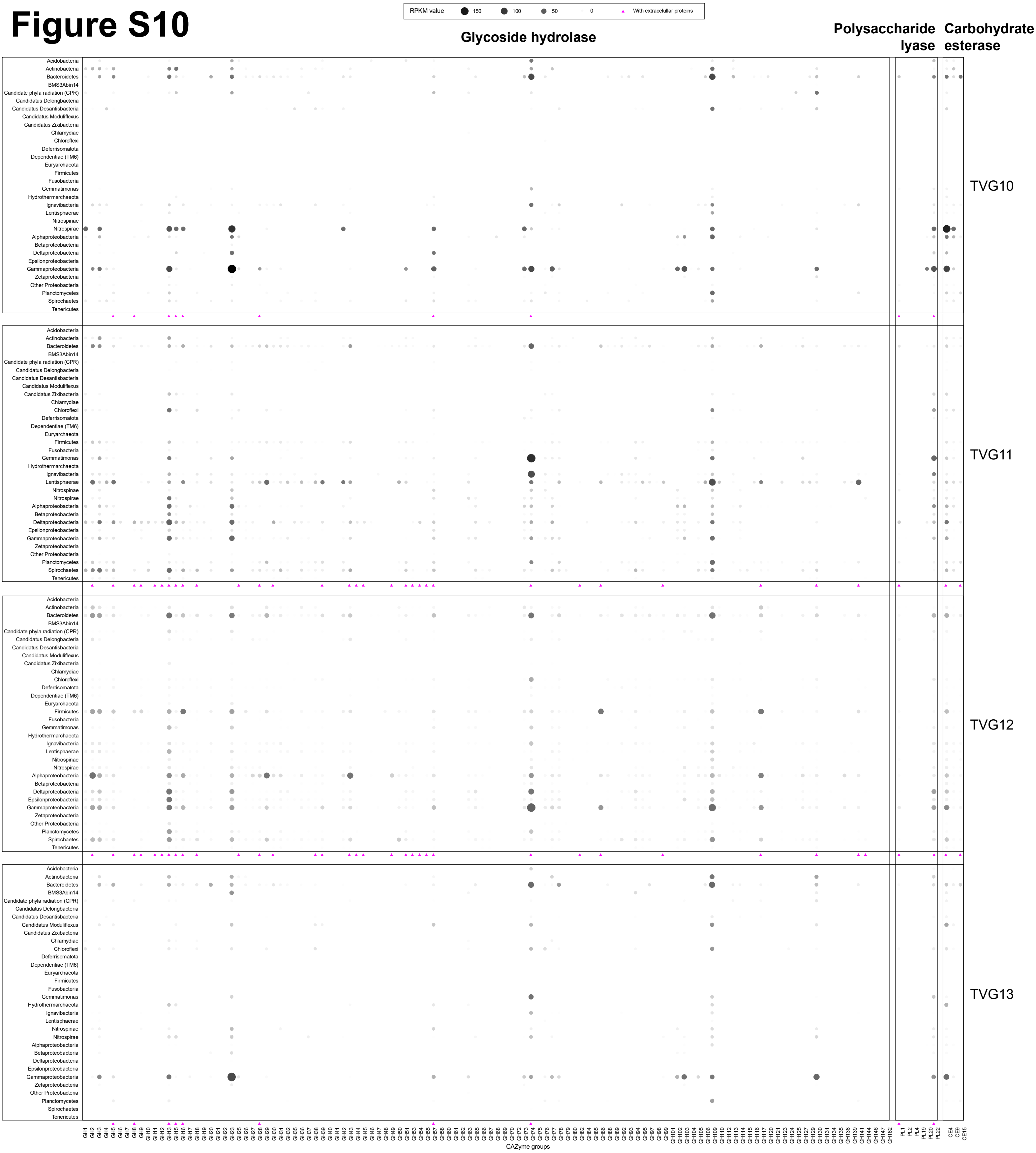
RPKM value
150
100
50
0
With extracelullar proteins
Glycoside hydrolase
Polysaccharide lyase
Carbohydrate esterase
Acidobacteria
Actinobacteria
Bacteroidetes
BMS3Abin14
Candidate phyla radiation (CPR)
Candidatus Delongbacteria
Candidatus Desantisbacteria
Candidatus Moduliflexus
Candidatus Zixibacteria
Chlamydiae
Chloroflexi
Deferrisomatota
Dependentiae (TM6)
Euryarchaeota
Firmicutes
Fusobacteria
Gemmatimonas
Hydrothermarchaeota
Ignavibacteria
Lentisphaerae
Nitrospinae
Nitrospirae
Alphaproteobacteria
Betaproteobacteria
Deltaproteobacteria
Epsilonproteobacteria
Gammaproteobacteria
Zetaproteobacteria
Other Proteobacteria
Planctomycetes
Spirochaetes
Tenericutes
TVG10
TVG11
TVG12
TVG13
GH1 GH2 GH3 GH4 GH5 GH6 GH7 GH8 GH9 GH10 GH11 GH12 GH13 GH15 GH16 GH17 GH18 GH19 GH20 GH21 GH22 GH23 GH25 GH26 GH27 GH28 GH29 GH30 GH31 GH32 GH35 GH36 GH37 GH38 GH39 GH40 GH41 GH42 GH43 GH44 GH45 GH46 GH47 GH48 GH49 GH50 GH51 GH53 GH54 GH55 GH57 GH58 GH60 GH61 GH62 GH63 GH65 GH66 GH67 GH68 GH69 GH70 GH72 GH73 GH74 GH75 GH76 GH77 GH78 GH79 GH80 GH82 GH84 GH85 GH86 GH88 GH89 GH92 GH93 GH94 GH95 GH97 GH98 GH99 GH101 GH102 GH103 GH104 GH105 GH106 GH109 GH110 GH112 GH113 GH114 GH115 GH116 GH117 GH120 GH121 GH123 GH124 GH125 GH127 GH129 GH130 GH131 GH134 GH135 GH138 GH139 GH141 GH144 GH146 GH147 GH162
PL1 PL2 PL4 PL19 PL20 PL22
CE4 CE9 CE15
CAZyme groups

Figure S11

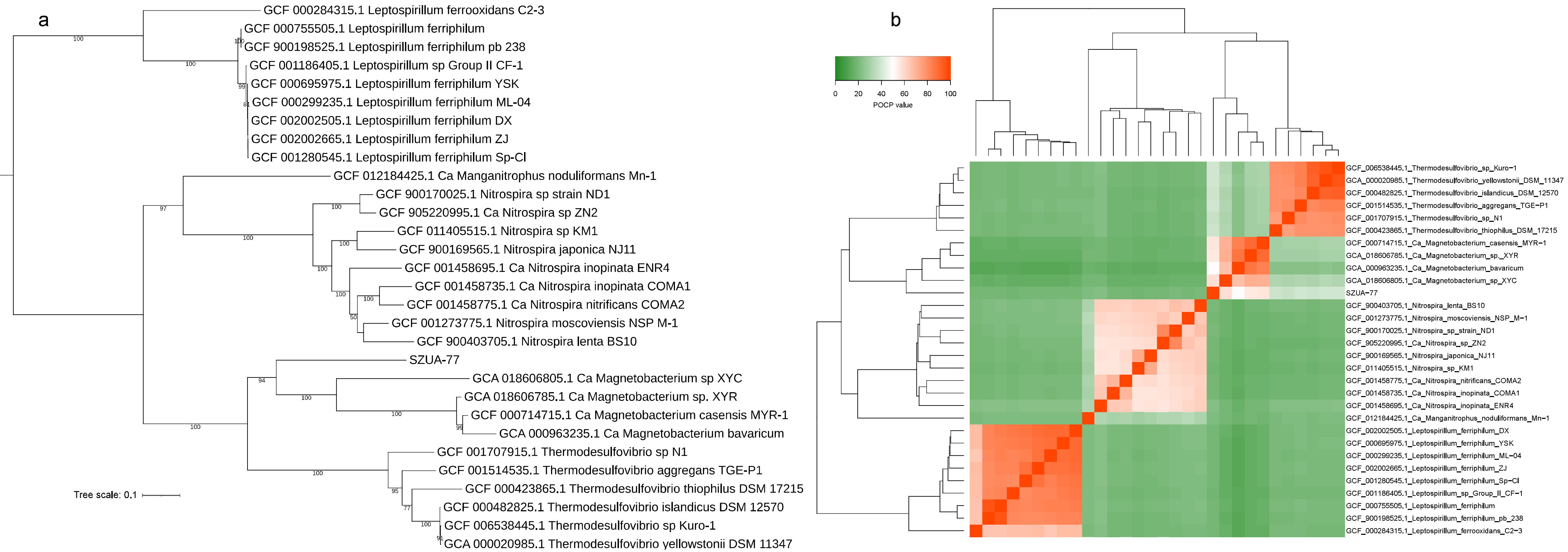
a
GCF 000284315.1 Leptospirillum ferrooxidans C2-3
GCF 000755505.1 Leptospirillum ferriphilum
GCF 900198525.1 Leptospirillum ferriphilum pb 238
GCF 001186405.1 Leptospirillum sp Group II CF-1
GCF 000695975.1 Leptospirillum ferriphilum YSK
GCF 000299235.1 Leptospirillum ferriphilum ML-04
GCF 002002505.1 Leptospirillum ferriphilum DX
GCF 002002665.1 Leptospirillum ferriphilum ZJ
GCF 001280545.1 Leptospirillum ferriphilum Sp-Cl
GCF 012184425.1 Ca Manganitrophus noduliformans Mn-1
GCF 900170025.1 Nitrospira sp strain ND1
GCF 905220995.1 Ca Nitrospira sp ZN2
GCF 011405515.1 Nitrospira sp KM1
GCF 900169565.1 Nitrospira japonica NJ11
GCF 001458695.1 Ca Nitrospira inopinata ENR4
GCF 001458735.1 Ca Nitrospira inopinata COMA1
GCF 001458775.1 Ca Nitrospira nitrificans COMA2
GCF 001273775.1 Nitrospira moscoviensis NSP M-1
GCF 900403705.1 Nitrospira lenta BS10
SZUA-77
GCA 018606805.1 Ca Magnetobacterium sp XYC
GCA 018606785.1 Ca Magnetobacterium sp. XYR
GCF 000714715.1 Ca Magnetobacterium casensis MYR-1
GCA 000963235.1 Ca Magnetobacterium bavaricum
GCF 001707915.1 Thermodesulfovibrio sp N1
GCF 001514535.1 Thermodesulfovibrio aggregans TGE-P1
GCF 000423865.1 Thermodesulfovibrio thiophilus DSM 17215
GCF 000482825.1 Thermodesulfovibrio islandicus DSM 12570
GCF 006538445.1 Thermodesulfovibrio sp Kuro-1
GCA 000020985.1 Thermodesulfovibrio yellowstonii DSM 11347
Tree scale: 0.1
b
0 20 40 60 80 100
POCP value
GCF_006538445.1_Thermodesulfovibrio_sp_Kuro−1
GCA_000020985.1_Thermodesulfovibrio_yellowstonii_DSM_11347
GCF_000482825.1_Thermodesulfovibrio_islandicus_DSM_12570
GCF_001514535.1_Thermodesulfovibrio_aggregans_TGE−P1
GCF_001707915.1_Thermodesulfovibrio_sp_N1
GCF_000423865.1_Thermodesulfovibrio_thiophilus_DSM_17215
GCF_000714715.1_Ca_Magnetobacterium_casensis_MYR−1
GCA_018606785.1_Ca_Magnetobacterium_sp._XYR
GCA_000963235.1_Ca_Magnetobacterium_bavaricum
GCA_018606805.1_Ca_Magnetobacterium_sp_XYC
SZUA−77
GCF_900403705.1_Nitrospira_lenta_BS10
GCF_001273775.1_Nitrospira_moscoviensis_NSP_M−1
GCF_900170025.1_Nitrospira_sp_strain_ND1
GCF_905220995.1_Ca_Nitrospira_sp_ZN2
GCF_900169565.1_Nitrospira_japonica_NJ11
GCF_011405515.1_Nitrospira_sp_KM1
GCF_001458775.1_Ca_Nitrospira_nitrificans_COMA2
GCF_001458735.1_Ca_Nitrospira_inopinata_COMA1
GCF_001458695.1_Ca_Nitrospira_inopinata_ENR4
GCF_012184425.1_Ca_Manganitrophus_noduliformans_Mn−1
GCF_002002505.1_Leptospirillum_ferriphilum_DX
GCF_000695975.1_Leptospirillum_ferriphilum_YSK
GCF_000299235.1_Leptospirillum_ferriphilum_ML−04
GCF_002002665.1_Leptospirillum_ferriphilum_ZJ
GCF_001280545.1_Leptospirillum_ferriphilum_Sp−Cl
GCF_001186405.1_Leptospirillum_sp_Group_II_CF−1
GCF_000755505.1_Leptospirillum_ferriphilum
GCF_900198525.1_Leptospirillum_ferriphilum_pb_238
GCF_000284315.1_Leptospirillum_ferrooxidans_C2−3

Figure S12

a

Methods	Command settings
bin1	--minContig 2500
bin2	--minContig 2500 --verysensitive
bin3	--minContig 1500
bin4	--minContig 1500 --verysensitive
bin5	--minContig 1500 --verysensitive -B 20
bin6	--minContig 1500 -B 20 --specific
bin7	--minContig 1500 -B 20 --specific --pB 20
bin8	--minContig 1500 -B 20 --specific --pB 5
bin9	--minContig 2500 --superspecific
bin10	--minContig 2500 --superspecific -B 20
bin11	--minContig 2500 --superspecific -B 20 --pB 20
bin12	--minContig 2500 --superspecific -B 20 --pB 5 --very

b

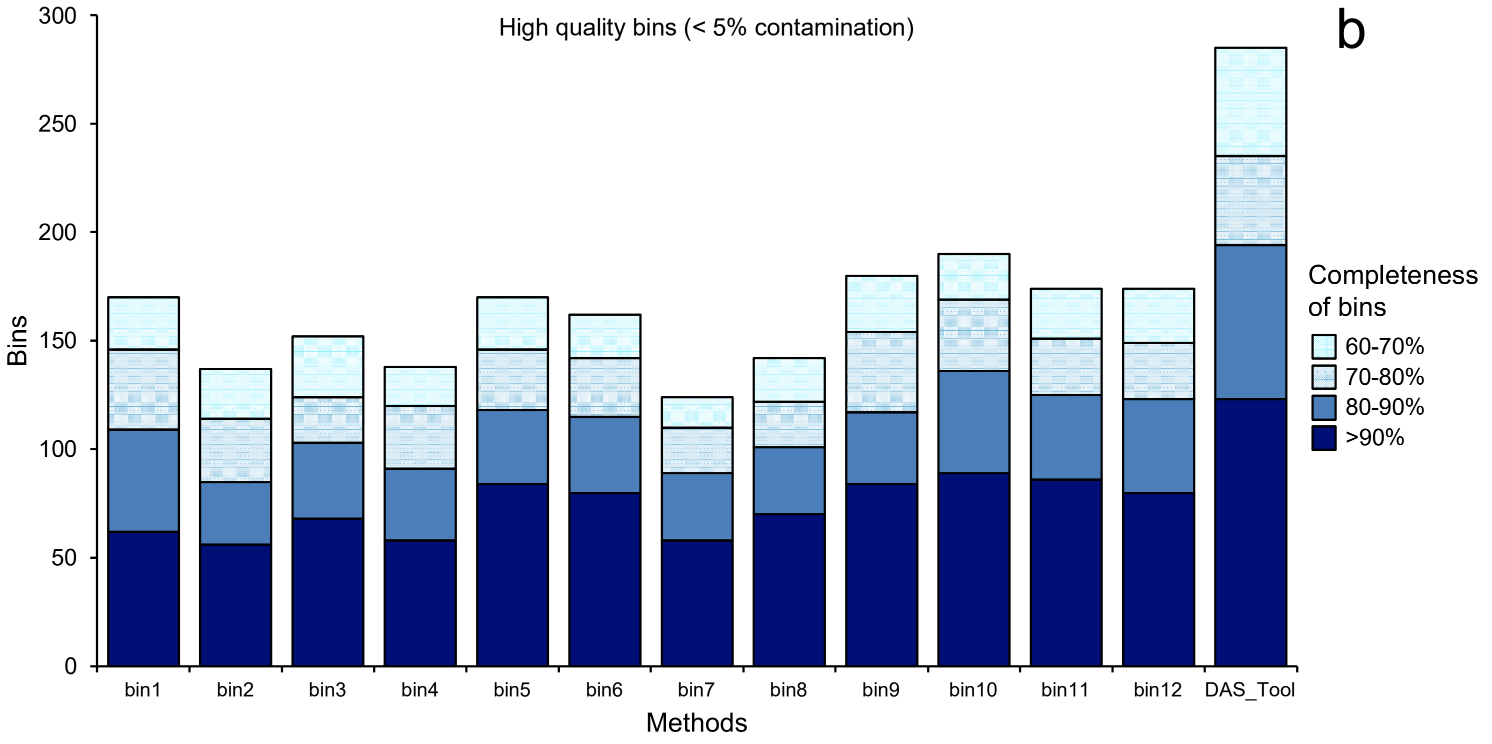

Figure S13

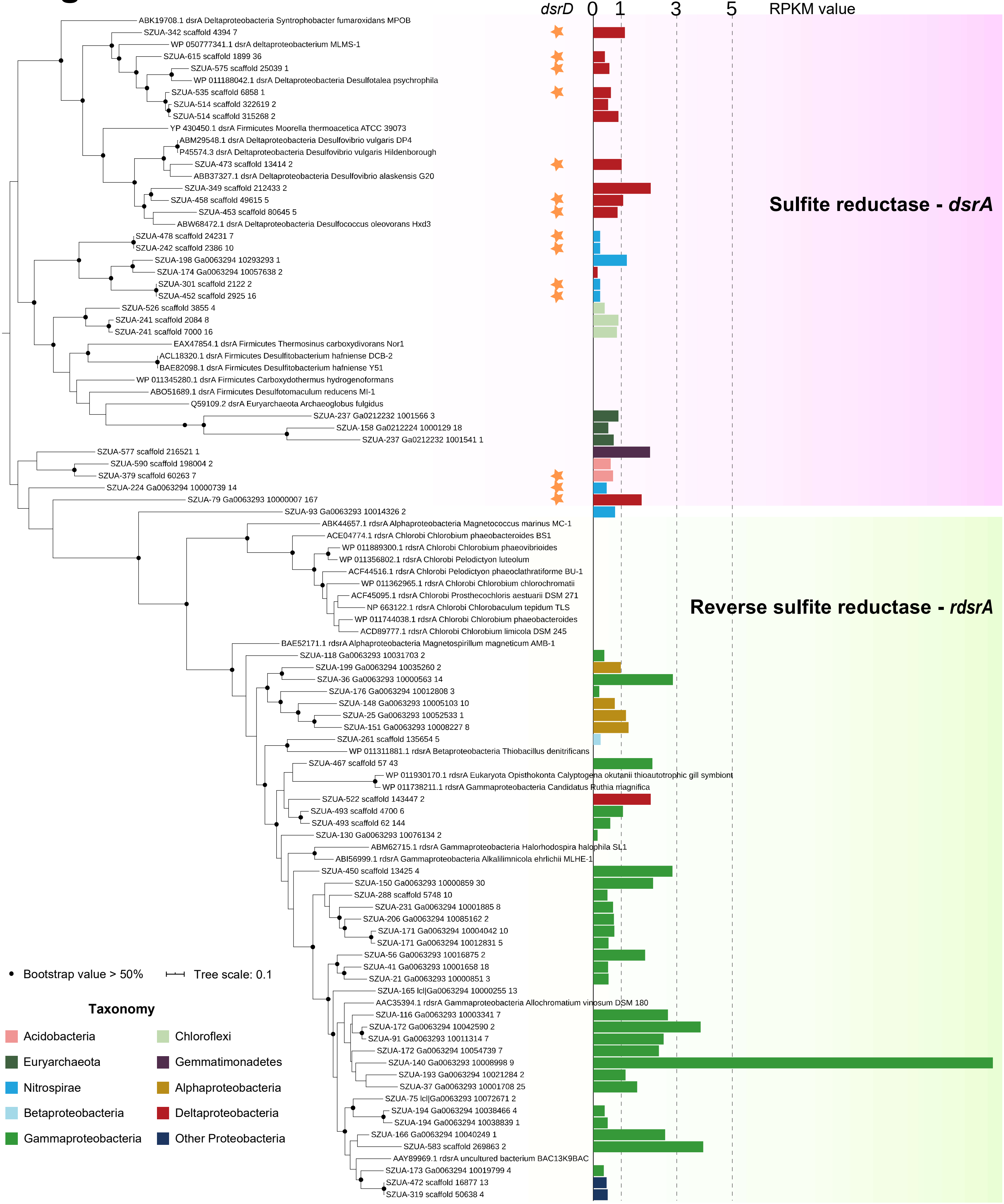

dsrD
0
1
3
5
RPKM value
Sulfite reductase - dsrA
Reverse sulfite reductase - rdsrA
ABK19708.1 dsrA Deltaproteobacteria Syntrophobacter fumaroxidans MPOB
SZUA-342 scaffold 4394 7
WP 050777341.1 dsrA deltaproteobacterium MLMS-1
SZUA-615 scaffold 1899 36
SZUA-575 scaffold 25039 1
WP 011188042.1 dsrA Deltaproteobacteria Desulfotalea psychrophila
SZUA-535 scaffold 6858 1
SZUA-514 scaffold 322619 2
SZUA-514 scaffold 315268 2
YP 430450.1 dsrA Firmicutes Moorella thermoacetica ATCC 39073
ABM29548.1 dsrA Deltaproteobacteria Desulfovibrio vulgaris DP4
P45574.3 dsrA Deltaproteobacteria Desulfovibrio vulgaris Hildenborough
SZUA-473 scaffold 13414 2
ABB37327.1 dsrA Deltaproteobacteria Desulfovibrio alaskensis G20
SZUA-349 scaffold 212433 2
SZUA-458 scaffold 49615 5
SZUA-453 scaffold 80645 5
ABW68472.1 dsrA Deltaproteobacteria Desulfococcus oleovorans Hxd3
SZUA-478 scaffold 24231 7
SZUA-242 scaffold 2386 10
SZUA-198 Ga0063294 10293293 1
SZUA-174 Ga0063294 10057638 2
SZUA-301 scaffold 2122 2
SZUA-452 scaffold 2925 16
SZUA-526 scaffold 3855 4
SZUA-241 scaffold 2084 8
SZUA-241 scaffold 7000 16
EAX47854.1 dsrA Firmicutes Thermosinus carboxydivorans Nor1
ACL18320.1 dsrA Firmicutes Desulfitobacterium hafniense DCB-2
BAE82098.1 dsrA Firmicutes Desulfitobacterium hafniense Y51
WP 011345280.1 dsrA Firmicutes Carboxydothermus hydrogenoformans
ABO51689.1 dsrA Firmicutes Desulfotomaculum reducens MI-1
Q59109.2 dsrA Euryarchaeota Archaeoglobus fulgidus
SZUA-237 Ga0212232 1001566 3
SZUA-158 Ga0212224 1000129 18
SZUA-237 Ga0212232 1001541 1
SZUA-577 scaffold 216521 1
SZUA-590 scaffold 198004 2
SZUA-379 scaffold 60263 7
SZUA-224 Ga0063294 10000739 14
SZUA-79 Ga0063293 10000007 167
SZUA-93 Ga0063293 10014326 2
ABK44657.1 rdsrA Alphaproteobacteria Magnetococcus marinus MC-1
ACE04774.1 rdsrA Chlorobi Chlorobium phaeobacteroides BS1
WP 011889300.1 rdsrA Chlorobi Chlorobium phaeovibrioides
WP 011356802.1 rdsrA Chlorobi Pelodictyon luteolum
ACF44516.1 rdsrA Chlorobi Pelodictyon phaeoclathratiforme BU-1
WP 011362965.1 rdsrA Chlorobi Chlorobium chlorochromatii
ACF45095.1 rdsrA Chlorobi Prosthecochloris aestuarii DSM 271
NP 663122.1 rdsrA Chlorobi Chlorobaculum tepidum TLS
WP 011744038.1 rdsrA Chlorobi Chlorobium phaeobacteroides
ACD89777.1 rdsrA Chlorobi Chlorobium limicola DSM 245
BAE52171.1 rdsrA Alphaproteobacteria Magnetospirillum magneticum AMB-1
SZUA-118 Ga0063293 10031703 2
SZUA-199 Ga0063294 10035260 2
SZUA-36 Ga0063293 10000563 14
SZUA-176 Ga0063294 10012808 3
SZUA-148 Ga0063293 10005103 10
SZUA-25 Ga0063293 10052533 1
SZUA-151 Ga0063293 10008227 8
SZUA-261 scaffold 135654 5
WP 011311881.1 rdsrA Betaproteobacteria Thiobacillus denitrificans
SZUA-467 scaffold 57 43
WP 011930170.1 rdsrA Eukaryota Opisthokonta Calyptogena okutanii thioautotrophic gill symbiont
WP 011738211.1 rdsrA Gammaproteobacteria Candidatus Ruthia magnifica
SZUA-522 scaffold 143447 2
SZUA-493 scaffold 4700 6
SZUA-493 scaffold 62 144
SZUA-130 Ga0063293 10076134 2
ABM62715.1 rdsrA Gammaproteobacteria Halorhodospira halophila SL1
ABI56999.1 rdsrA Gammaproteobacteria Alkalilimnicola ehrlichii MLHE-1
SZUA-450 scaffold 13425 4
SZUA-150 Ga0063293 10000859 30
SZUA-288 scaffold 5748 10
SZUA-231 Ga0063294 10001885 8
SZUA-206 Ga0063294 10085162 2
SZUA-171 Ga0063294 10004042 10
SZUA-171 Ga0063294 10012831 5
SZUA-56 Ga0063293 10016875 2
SZUA-41 Ga0063293 10001658 18
SZUA-21 Ga0063293 10000851 3
SZUA-165 lcl|Ga0063294 10000255 13
AAC35394.1 rdsrA Gammaproteobacteria Allochromatium vinosum DSM 180
SZUA-116 Ga0063293 10003341 7
SZUA-172 Ga0063294 10042590 2
SZUA-91 Ga0063293 10011314 7
SZUA-172 Ga0063294 10054739 7
SZUA-140 Ga0063293 10008998 9
SZUA-193 Ga0063294 10021284 2
SZUA-37 Ga0063293 10001708 25
SZUA-75 lcl|Ga0063293 10072671 2
SZUA-194 Ga0063294 10038466 4
SZUA-194 Ga0063294 10038839 1
SZUA-166 Ga0063294 10040249 1
SZUA-583 scaffold 269863 2
AAY89969.1 rdsrA uncultured bacterium BAC13K9BAC
SZUA-173 Ga0063294 10019799 4
SZUA-472 scaffold 16877 13
SZUA-319 scaffold 50638 4
Bootstrap value > 50%
Tree scale: 0.1
Taxonomy
Acidobacteria
Chloroflexi
Euryarchaeota
Gemmatimonadetes
Nitrospirae
Alphaproteobacteria
Betaproteobacteria
Deltaproteobacteria
Gammaproteobacteria
Other Proteobacteria
